# Reactivation of the Photosynthetic Apparatus of Resurrection Plant *Haberlea rhodopensis* during the Early Phase of Recovery from Drought- and Freezing-Induced Desiccation

**DOI:** 10.3390/plants11172185

**Published:** 2022-08-23

**Authors:** Gergana Mihailova, Nikolai K. Christov, Éva Sárvári, Ádám Solti, Richard Hembrom, Katalin Solymosi, Áron Keresztes, Maya Velitchkova, Antoaneta V. Popova, Lyudmila Simova-Stoilova, Elena Todorovska, Katya Georgieva

**Affiliations:** 1 Institute of Plant Physiology and Genetics, Bulgarian Academy of Sciences, Academic Georgi Bonchev Str., Bilding 21, 1113 Sofia, Bulgaria; 2 AgroBioInstitute, Agricultural Academy, 8 Dragan Tsankov Blvd., 1164 Sofia, Bulgaria; 3 Department of Plant Physiology and Molecular Plant Biology, Institute of Biology, Faculty of Science, ELTE Eötvös Loránd University, Pázmány Péter Sétány 1/C, H-1117 Budapest, Hungary; 4 Department of Plant Anatomy, Institute of Biology, Faculty of Science, ELTE Eötvös Loránd University, Pázmány Péter Sétány 1/C, H-1117 Budapest, Hungary; 5 Institute of Biophysics and Biomedical Engineering, Bulgarian Academy of Sciences, Academic Georgi Bonchev Str., Bilding 21, 1113 Sofia, Bulgaria

**Keywords:** rehydration, drought, low temperature, cell ultrastructure, pigment–protein complexes, photosynthetic proteins, ELIP, dehydrins, proteases, gene expression

## Abstract

*Haberlea rhodopensis* is a unique desiccation-tolerant angiosperm that also survives winter frost. As, upon freezing temperatures, *H. rhodopensis* desiccates, the taxon is proposed to survive low temperature stress using its desiccation tolerance mechanisms. To reveal the validity of this hypothesis, we analyzed the structural alterations and organization of photosynthetic apparatus during the first hours of recovery after drought- and freezing-induced desiccation. The dynamics of the ultrastructure remodeling in the mesophyll cells and the restoration of the thylakoid membranes shared similarities independent of the reason for desiccation. Among the most obvious changes in thylakoid complexes, the proportion of the PSI-LHCII complex strongly increased around 70% relative water content (RWC), whereas the proportion of Lhc monomers decreased from the beginning of rehydration. We identified enhanced levels of cyt *b_6_f* complex proteins that contributed to the enhanced electron flow. The high abundance of proteins related to excitation energy dissipation, PsbS, Lhcb5, Lhcb6 and ELIPs, together with the increased content of dehydrins contributed to the preservation of cellular integrity. *ELIP* expression was maintained at high levels up to 9 h into recovery. Although the recovery processes from drought- and freezing-induced desiccation were found to be similar in progress and time scale, slight variations indicate that they are not identical.

## 1. Introduction

Environmental extremities such as frost and drought are challenging to plant life as well as agricultural production [1]. Periods of drought are constantly increasing due to climate change and global warming. Thus, understanding the mechanisms that enable plants to cope with limited water is increasingly important. With their ability to survive desiccation to an air-dry state, resurrection plants represent a promising model system for studying the mechanisms of drought tolerance and for the identification of genes that could potentially enhance the drought tolerance of crops through biotechnological approaches [2,3,4]. Nevertheless, the majority of desiccation-tolerant plant species inhabit subtropical to tropical regions with little risk of low-temperature stress [5]. Plants that are adapted to temperate climates can increase their freezing tolerance once exposed to low, non-freezing temperatures. The homoiochlorophyllous resurrection plant *H. rhodopensis* is unique among angiosperm resurrection plants in its ability to survive subzero temperatures. Like drought, freezing stress also affects the availability of liquid water and thus causes the desiccation of plants and corresponding ultrastructural changes in mesophyll cells [6].

Photosynthesis is one of the primary processes affected by both drought and frost stress. Upon drought, photosynthesis inhibition occurs in both desiccation-tolerant and desiccation-sensitive higher plants [7,8]. Stomatal closure, pigment degradation or destruction of the photosynthetic apparatus contribute to the loss of photosynthetic capacity upon drought stress [9]. Like drought, low temperature stress also affects photosynthesis, primarily in the balance between the photochemical and biochemical reactions. Imbalance induces changes in photosystem II (PSII) excitation pressure that also reflects the redox state of the thylakoidal plastoquinone pool [10,11]. Finally, perturbed operation of the electron transport chain results in the formation of reactive oxygen species (ROS) and oxidative damages.

In resurrection plants, downregulation of photosynthesis during dehydration is achieved by one of the two mechanisms termed poikilochlorophylly and homoiochlorophylly [12]. The homoiochlorophyllous desiccation-tolerant (HDT) plants retain their photosynthetic apparatus and chlorophylls during drying, and they recover faster after rehydration. Thylakoid pigment–protein complexes have shown high stability during desiccation in *H. rhodopensis* [13] and *Boea hygrometrica* [9]. Nevertheless, complete inhibition of photosynthetic activity in the air-dry state is accompanied by some changes in the amount of essential photosynthetic proteins [14,15,16,17]. Previously, we showed that in *H. rhodopensis* the contents of PSI reaction center proteins, PsaA/B, was less affected by desiccation than that of PSII, PsbA (D1) and PsbD (D2), which is consistent with observations of higher decline in the photochemical activity of PSII compared to PSI [18]. Regarding the light harvesting antennae (LHC), a slight increase was reported in the LHCII proteins of desiccated HDT plants [14,17]. Nevertheless, it should be noted that changes in the amounts of photosynthetic proteins during desiccation are highly dependent upon experimental conditions, especially on light intensity, as shown in the HDT taxon *Craterostigma plantagineum* [19]. Significant reduction in the levels of cytochrome *f* (cyt *f*) protein was found in partially dehydrated *C. pumilum* plants, and the key role of the cyt *b_6_f* complex in inhibiting photosynthetic electron transport during dehydration was suggested [17]. The content of the 33 kDa protein in the oxygen-evolving complex, among the most sensitive parts of PSII, got reduced in moderately dehydrated *H. rhodopensis* plants [16], whereas the 23 kDa protein of this complex was shown to accumulate in the dehydrated leaves of *B. hygrometrica* [20]. During drought- and freezing-induced desiccation, the quantity of photosynthetic proteins slightly differed in moderately desiccated leaves, while severe desiccation induced similar changes in *H. rhodopensis* [6]. In addition to the complex organization of pigment–protein complexes, physicochemical properties, especially the fluidity of the lipid matrix of thylakoids, are essential for maintaining the structural and functional integrity of main photosynthetic complexes. It has been shown that during the dehydration of *C. pumilum* the alteration of pigment–protein complexes was accompanied by changes to the lipid matrix—the formation of an inverted hexagonal phase was observed [17]. Since fluidity of the lipid matrix is crucial for structural rearrangements and the translocation of protein complexes, it has a deep impact on the tolerance to unfavorable environments.

HDT resurrection plants require active protective mechanisms during desiccation to maintain the structural integrity of the photosynthetic apparatus and to overcome oxidative damage [21]. Multiple studies pointed out the accumulation of non-enzymatic antioxidants [22,23,24] and enhanced activity of antioxidant enzymes during the desiccation of HDT taxa [25,26,27]. In addition to increased antioxidative protection, a rearrangement of the cell content also characterizes desiccation [28], where an increase in the leaf sucrose content was associated with the formation of secondary vacuoles in *H. rhodopensis* [28]. Parallel to the degree of desiccation, thylakoids tend to be arranged concentrically without any damage to their integrity [29,30].

Stress-induced proteins like early light-induced proteins (ELIPs), late embryogenesis-abundant (LEA) proteins, and small heat shock proteins (HSPs) also contribute to preserving the integrity of cellular constituents during dehydration. ELIPs are pigment-binding proteins which protect the photosynthetic apparatus against photo-oxidative damage. Alamillo and Bartels [19] found that ELIP-like desiccation-induced protein DSP22 accumulates in the thylakoid membranes of *C. plantagineum* in response to desiccation. It has been suggested that by binding zeaxanthin and chlorophylls, ELIPs contribute to the increased dissipation of excess absorbed energy through non-photochemical quenching [31] and to maintaining free pigments at low levels under stress conditions [32]. LEA 2 group proteins, or so-called dehydrins, also accumulate in high amounts in the vegetative tissues, especially in the cytoplasm and chloroplasts of mesophyll cells of resurrection plants in response to water deficit [33,34,35,36]. Layton et al. [37] proposed that dehydrins enable reversible, large cell-wall deformation thus avoiding mechanical failure during drought. They protect proteins against denaturation, stabilize membranes through ion sequestration and the replacement of hydrogen bonding, and they may interact with sugars promoting vitrification [34,38,39].

Although the mechanisms that enable resurrection plants to cope with severe drought are well studied, the most significant difference between drought-sensitive and resurrection plants, a difference which is outside the focus of multiple studies, is the ability of the latter group to recover from the air-dry stage. The recovery of the relative water content (RWC) and photosynthetic activity has already been demonstrated in *H. rhodopensis*. After one day of rehydration, the physiological status of desiccated plants improved significantly, and after 7 days’ (d) time, it returned to a similar state as that of well-hydrated plants [40]. However, the dynamics of the recovery process, especially during the initiation of rehydration, have not been mapped yet. Initial processes have special importance since rehydration from the air-dry state also represents a stress to plant tissues, similar to dehydration. As, upon freezing temperatures, *H. rhodopensis* also desiccates, the taxon is proposed to survive low temperature stress using its desiccation tolerance mechanisms [6], which suggests a high degree of similarity in the processes and the time scale of the alterations upon the start of the recovery. 

Thus, we aimed to analyze and compare the alterations in leaf structure and rearrangements of the photosynthetic apparatus of the HDT plant *H. rhodopensis* during the early phase of recovery from drought- and freezing-induced desiccation. The contribution of stress-induced proteins, among other ELIPs and dehydrins, to plant recovery and the changes in the relative transcript abundance of some *ELIP* genes were determined. 

## 2. Results

### 2.1. Alteration in the Structure upon Recovery

**Figure 1:**
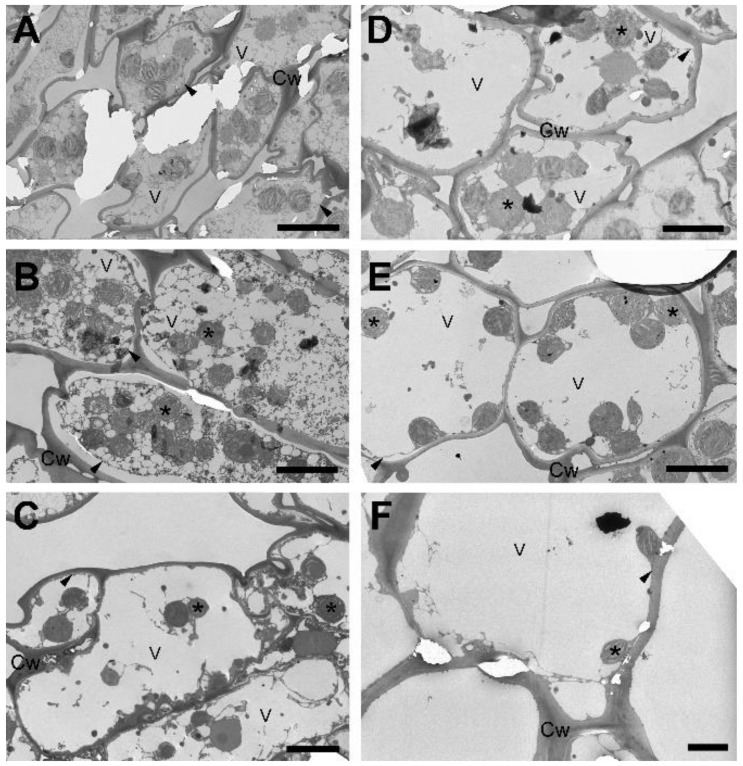
Cell ultrastructure during recovery from drought-induced desiccation (RAD) of *Haberlea rhodopensis* leaves. (**A**): 0 h, (**B**): 9 h, (**C**): 15 h, (**D**): 18 h, (**E**): 24 h, (**F**): 30 h after rehydration. Arrowhead: plasma membrane; asterisk: plastid; Cw: cell wall; V: vacuole. Scale bar is equal to 10 μm.

**Figure 2:**
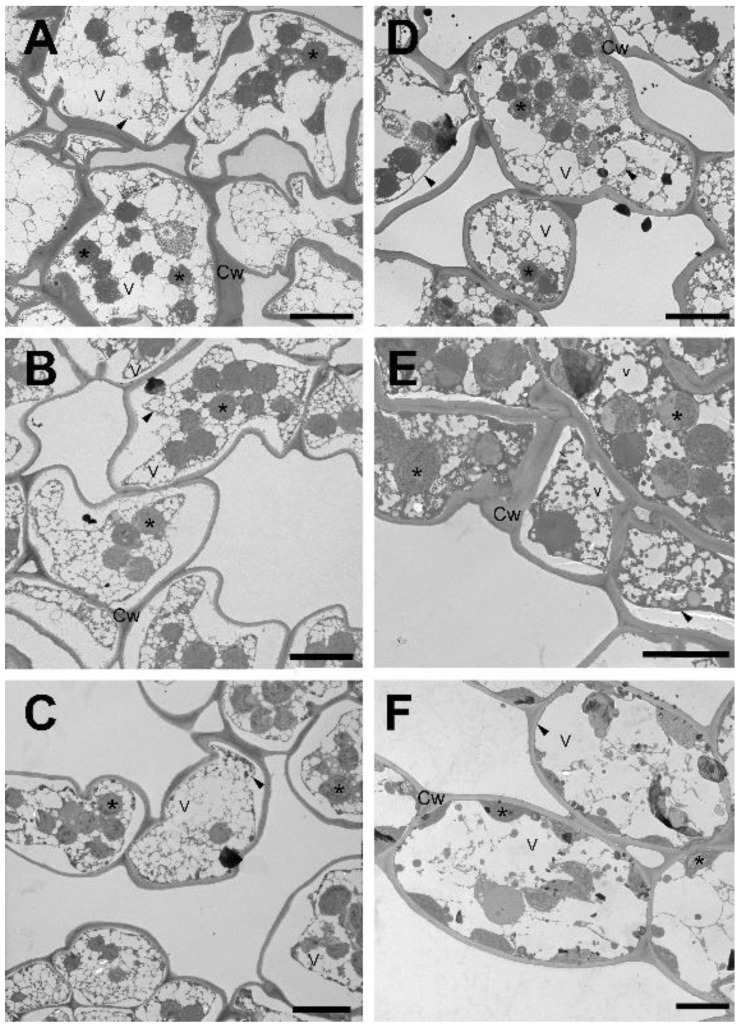
Cell ultrastructure during recovery from freezing-induced desiccation (RAF) of *H. rhodopensis* leaves. (**A**): 0 h, (**B**): 1 h, (**C**): 5 h, (**D**): 9 h, (**E**): 15 h, (**F**): 24 h after rehydration. Arrowhead: plasma membrane; asterisk: plastid; Cw: cell wall; V: vacuole. Scale bar is equal to 10 μm.

Both drought- and freezing-induced desiccation cause massive alterations to the ultrastructure of mesophyll cells. To reveal the time course of restoration of the ultrastructure to a state resembling the well-hydrated stage, we investigated these processes during the rehydration of mesophyll cells after drought- (RAD) and freezing-induced (RAF) desiccation at low magnification using transmission electron microscopy (TEM) focusing on the initial hours of rehydration. In the first 9 h (h) of rehydration, the rounded chloroplasts started to relocate from the cell interior back to the plasmalemma, and the first plastids were observed to be located along the plasma membrane by the end of this time period (Figure 1A,B and Figure 2A–E). During the first 9 h of recovery, a number of small vacuoles were continuously visible in the cell interior, separated from each other by a cytoplasmic network. As a next phase of rearrangement, the small vacuoles were gradually replaced by few or a single large vacuole, while most plastids reached the plasmalemma (Figure 1C–E and Figure 2E). Finally, in the third phase, the chloroplasts regained their half-lens shape (Table 1) and came in full contact with the plasma membrane (Figure 1F and Figure 2F). This process was completed within 24 h of rehydration during RAF and within 30 h during RAD.

**Figure 3:**
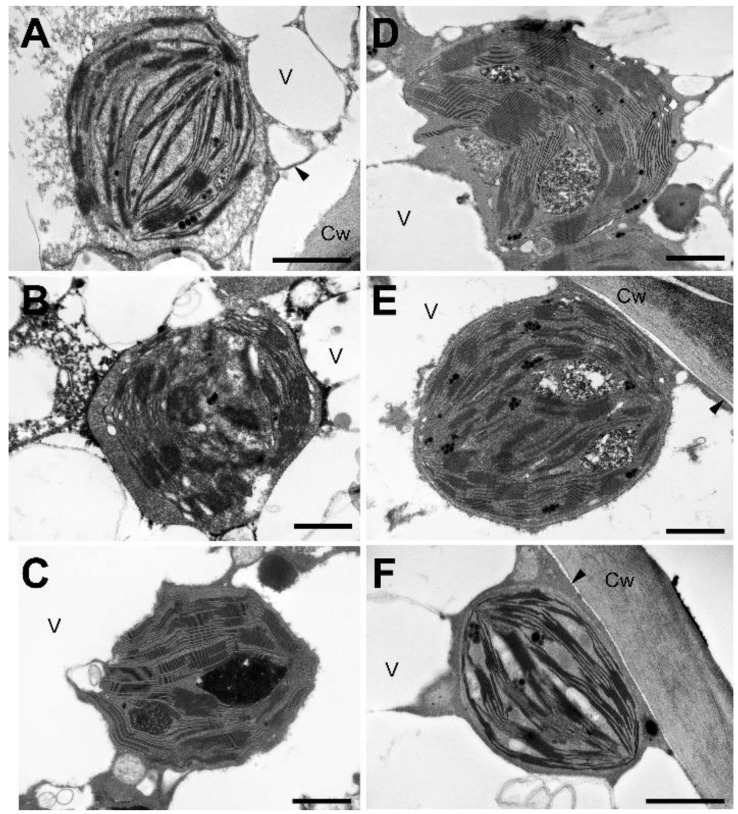
Chloroplast ultrastructure during recovery from drought-induced desiccation (RAD) of *H. rhodopensis* leaves. (**A**): 0 h, (**B**): 9 h, (**C**): 15 h, (**D**): 18 h, (**E**): 24 h, and (**F**): 30 h after rehydration. Arrowhead: plasma membrane; Cw: cell wall; V: vacuole. Scale bar is equal to 1 μm.

**Figure 4:**
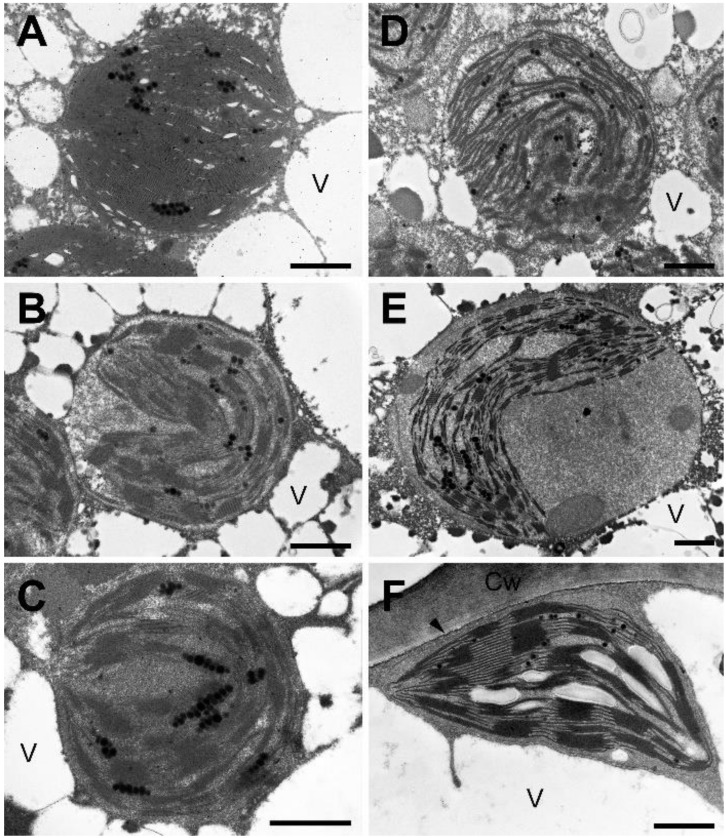
Chloroplast ultrastructure during recovery from freezing-induced desiccation (RAF) of *H. rhodopensis* leaves. (**A**): 0 h, (**B**): 1 h, (**C**): 5 h, (**D**): 9 h, (**E**): 15 h, and (**F**): 24 h after rehydration. Arrowhead: plasma membrane; Cw: cell wall; V: vacuole. Scale bar is equal to 1 μm.

Observation of the inner structure of the chloroplasts (Figure 3 and Figure 4) at high magnification indicated well visible grana and stroma lamellae in the desiccated stage (0 h; Figure 3A and Figure 4A). In the desiccated stage, the lamellar system accommodated to the roundish shape of chloroplasts either by forming arches on both sides of the central part of the stroma matrix (Figure 3E and Figure 4C), or by attaining a cup-shaped arrangement (Figure 3D and Figure 4B,E). In the desiccated stage, but also during RAD and RAF, small electron-dense plastoglobules were present. Additionally, large, membrane-bound, electron-dense or flocculent inclusions occurred in the early phases of RAD only (Figure 3C–E). Comparison of the granum repeat distance (RD) values in the fully desiccated samples and in samples taken 24 h after initiating RAF showed no significant differences during rehydration (Table 1). On the other hand, the low RD values present in the fully desiccated stage were significantly increased during 24 h rehydration under RAD (Table 1).

### 2.2. Reorganization of Pigment–Protein Complexes during Recovery

**Figure 5:**
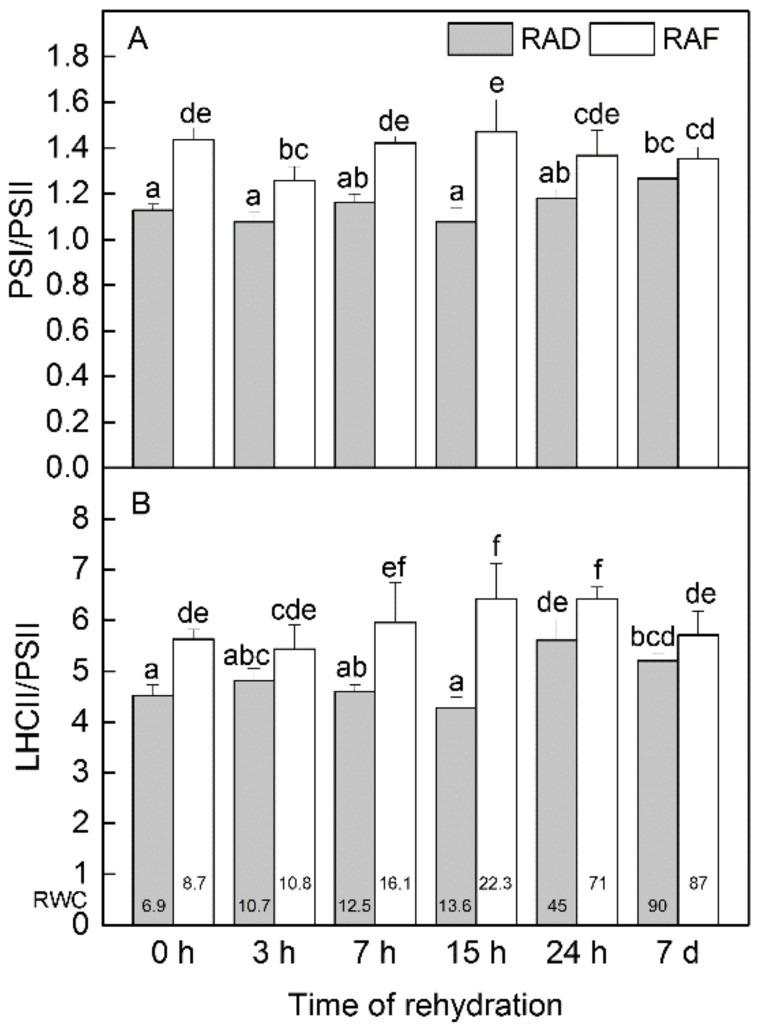
Changes in the ratios of the main thylakoid complexes PSI/PSII (**A**) and LHCII/PSII (**B**) in *H. rhodopensis* during recovery from drought—(RAD) and freezing-induced desiccation (RAF) as a function of the time of rehydration. Thylakoids (500 µg chlorophyll mL^−1^) were solubilised using 1% (*w*/*V*) *β*-DM plus 1% (*w*/*V*) digitonin, and complexes were separated in 4.3–12% Blue Native gel gradients. PS—photosystem; LHCII—light-harvesting complex II. The ratios were calculated from 1.D BN bands, i.e., PSI and PSII contain the bound antennae, and LHCII represents free complexes. Values are given as mean ± SE. Changes between RAD and RAF were statistically compared. The same letters within a graph indicate no significant differences assessed by the Fisher LSD test (*p* ≤ 0.05) after performing ANOVA. The RWC of plants at each time point, presented in %, are shown at the bottom of the columns.

**Figure 6:**
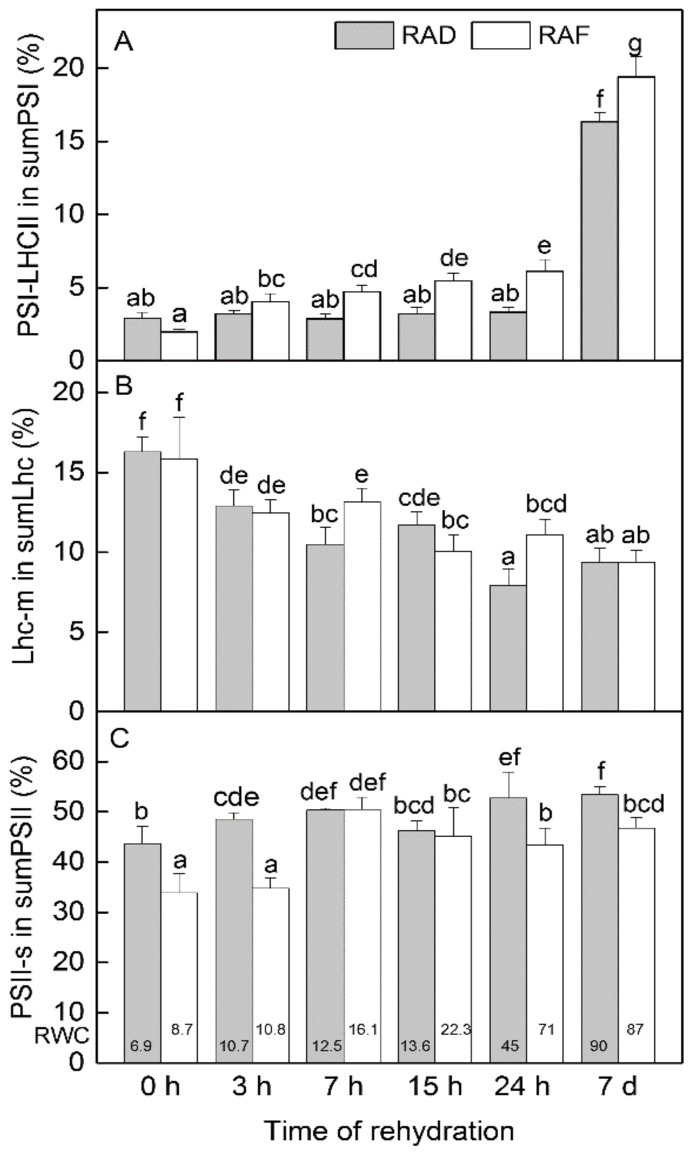
Changes in the proportion of the given assembly forms of thylakoid complexes in *H. rhodopensis* during the recovery from drought—(RAD) and freezing-induced desiccation (RAF) as a function of the time of rehydration. (**A**) Percentage of PSI-LHCII assembly form/band in the sum of all PSI forms/bands (PSI-megacomplex + PSI-LHCII + PSI + PSI-core = 100%); (**B**) Percentage of Lhc-m in sum free Lhc (LHCII-a + LHCII-t + Lhc-m = 100%); (**C**) Percentage of PSII-(mega-) and supercomplexes in sum PSII ((PSII-mega) + PSII-s + PSII-d + PSII-m = 100%). Thylakoids (500 µg chlorophyll mL^−1^) were solubilized using 1% (*w*/*V*) *β*-DM plus 1% (*w*/*V*) digitonin, and complexes were separated in 4.3–12% Blue Native gel gradients. PS—photosystem; LHC/Lhc—light-harvesting complex; s—supercomplex; m—monomer. Values are given as mean ± SE. Changes between RAD and RAF were statistically compared. The same letters within a graph indicate no significant differences assessed by the Fisher LSD test (*p* ≤ 0.05) after performing ANOVA. The RWC of plants at each time point, presented in %, are shown at the bottom of the columns.

The complete recovery of the thylakoid composition of *H. rhodopensis* from both drought- and freezing-induced desiccation required multiple days in total. Investigating the recovery up to 7 days in detail, the increase in PSI-LHCII content was the most conspicuous change in the gel patterns under both RAD and RAF (Figures S1 and S2). Focusing on qualitative alterations during the recovery periods, the PSI/PSII ratio did not significantly change, while the LHCII/PSII ratio showed some elevation after 7 h under RAF and 24 h under RAD (Figure 5). Regarding the reorganization of the assembly forms of complexes, the increase in the proportion of PSI-LHCII complex happened continuously under RAF but was observable only after 7 days under RAD (Figure 6A). However, this difference seemed to be in connection with the slower rise in RWC under RAD, which was indicated at the bottom of the columns for each time point. The proportion of Lhc-m decreased from the beginning of the recovery period in both treatments (Figure 6B). This was accompanied by an increased proportion of LHCII-t, while that of LHCII-a did not change. The reorganization of PSII-s complexes was recorded around 7 h of rehydration under RAF, while it was more pronounced at 24 h under RAD (Figure 6C). Increase in the relative amounts of PSII-s complexes started at around 25–30% RWC.

### 2.3. Changes in Photosynthetic Protein Abundance throughout Recovery

Regarding the alterations in the levels of photosynthetic proteins during early hours of RAD and RAF of *H. rhodopensis*, 3–15 h after the initiation of the rehydration were characterized by enhanced protein content during RAD and reduced protein content during RAF in regard to most of the proteins studied (Table 2 and Table 3; Figure S3). The content of PSII reaction center protein D1 gradually increased during both RAD and RAF, whereas no significant changes in the content of the heterodimer PSII reaction center protein member D2 were found during RAD. In turn, D2 content decreased from 3 up to 7 h after the initiation of recovery under RAF. The abundance of the inner antenna proteins of PSII, PsbC and PsbB, and PSI reaction center proteins, PsaA and PsaB, demonstrated identical tendency of enhanced or reduced abundance in the 3 to 7 h time frame after the initiation of recovery under RAD and RAF, respectively, and these changes were much more pronounced for PsbC and PsbB. The amount of the 33 kDa member of oxygen-evolving complex of PSII (PsbO) slightly decreased in the first hours of recovery, up to 15 and 7 h of RAD and RAF, respectively. Rehydration also led to accumulation of PsbS in all samples, both under RAD and RAF. Regarding cyt *b*_6_*f* complex components cyt *f*, cyt *b*_6_ and Rieske protein, alterations in their contents showed a similar trend of enhancement in the 3 to 15 h time frame after the initiation of recovery both under RAD and RAF, but more pronouncedly under RAD.

Neither RAD nor RAF affected the amount of LHC proteins significantly (Table 2 and Table 3; Figure S3). Among LHCII antenna proteins, the contents of Lhcb2, Lhcb4 (only under RAD), and Lhcb1 (only under RAF) increased during the entire course of recovery, while the amounts of Lhcb3 and Lhcb4 remained almost unchanged with a small decline in their content at 7 and 15 h both under RAD and RAF. The abundance of Lhcb1 (only under RAD), Lhcb5, and Lhcb6 (only under RAF) gradually decreased during the process of rehydration, declining to 60–80% of their content represented in the corresponding desiccated plants. PSI antenna complex LHCI exhibited minor changes in its amount compared to LHCII during recovery. Lhca1 and Lhca3 were characterized by a small increase in their content, whereas Lhca2 and Lhca4 were characterized by a slight decrease during rehydration.

### 2.4. Effect of Rehydration on the Fluidity of the Lipid Matrix of the Thylakoid Membranes

**Figure 7:**
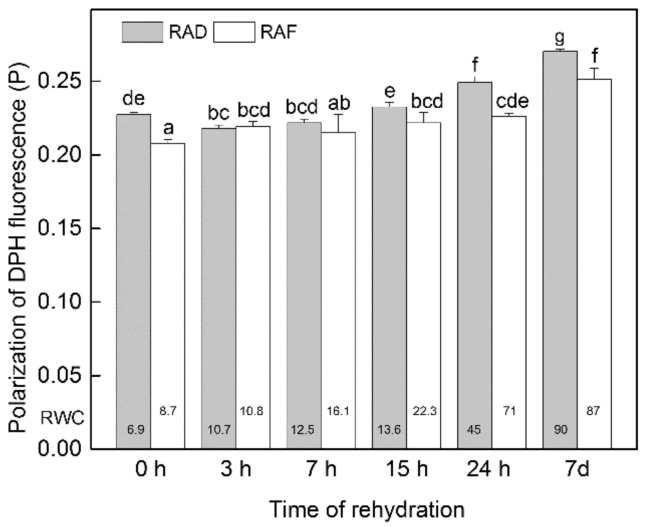
Changes in the fluidity of thylakoid membranes isolated from *H. rhodopensis* under recovery from drought- (RAD) and freezing-induced desiccation (RAF). Values are given as mean ± SE. Changes between RAD and RAF were statistically compared. The same letters within a graph indicate no significant differences assessed by the Fisher LSD test (*p* ≤ 0.05) after performing ANOVA. The RWC of plants at each time point, presented in %, are shown at the bottom of the columns.

The fluidity of the thylakoid lipid matrix was measured as the polarization degree of DPH (P) integrated into isolated *H. rhodopensis* thylakoids (Figure 7). The fluidity of the lipid phase in thylakoids isolated from plants desiccated by freezing was higher, indicated by lower P value (0.208 ± 0.003) and thus a lower-ordered lipid environment in comparison to plants desiccated by drought (0.227 ± 0.001). For the first few hours (3 and 7 h) under both RAF and RAD, no significant alterations in the lipid order in thylakoid membranes were registered. Nevertheless, after 15 and 24 h of rehydration, a gradual increase of P was detected, indicating an increase of the lipid order both under RAF and RAD. The degree of lipid order was further increased in thylakoid membranes by the 7th day of recovery both under RAF and RAD, reaching P values of 0.270 ± 0.001 and 0.252 ± 0.007, respectively. In consequence, the decrease in the fluidity of the thylakoids by the end of the 7 d recovery period was 19% and 21% under RAD and RAF, respectively, compared to the corresponding dried samples.

### 2.5. Protease Activity during Rehydration

**Figure 8:**
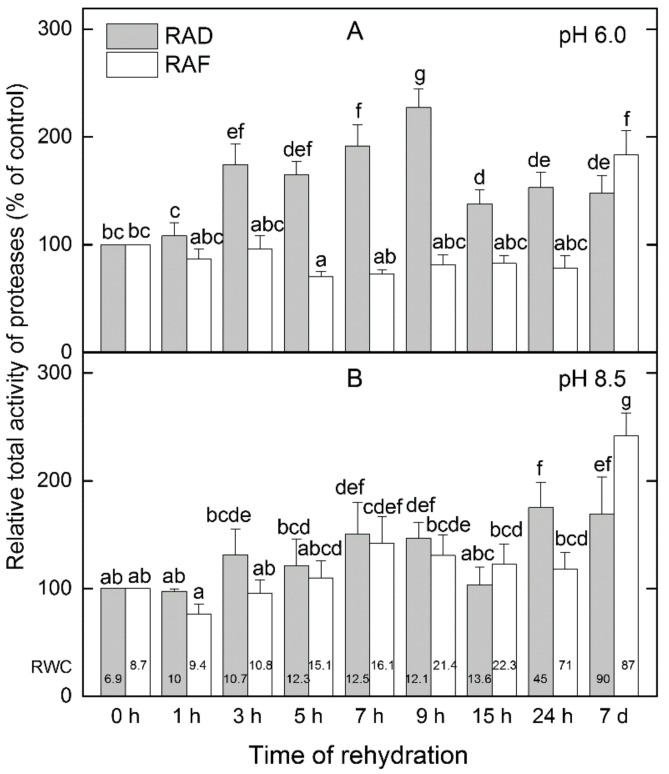
Relative proteolytic activity based on in-gel staining at pH 6.0 (**A**) and pH 8.5 (**B**) during the first hours of recovery of *H. rhodopensis* from drought- (RAD) and freezing-induced desiccation (RAF) expressed as percentage of desiccated plants (0 h). Data represent the mean of *n* = 3 ± SD. Changes between RAD and RAF were statistically compared. The same letters within a graph indicate no significant differences assessed by the Fisher LSD test (*p* ≤ 0.05) after performing ANOVA. The RWC of plants at each time point, presented in %, are shown at the bottom of the columns.

Using in-gel staining for protease activity at two preliminarily established pH optima (pH 6.0 and pH 8.5) 7–8 distinct activity bands in leaf extracts were shown. In general, the freezing-induced desiccation stage (0 h) was represented by higher proteolytic activity compared to drought-induced desiccation (Figure S4). Under RAF and RAD, alterations in the total protease activity were registered (Figure 8). Under RAF, we measured a diminution of the protease activity during the first hours of recovery that was most expressed at 5 h after the start of rehydration at pH 6.0, but only 1 h after the initiation of rehydration at pH 8.5 (total activity 70% and 76% of the corresponding desiccated samples, respectively). In comparison, a general increase in the protease activity was revealed under RAD where the highest activities were recorded at 9 h at pH 6.0 and at 24 h at pH 8.5 (total activity of 227% and 175% of the corresponding desiccated samples, respectively). High mobility bands were detected in samples rehydrated for 24 h and 7 d. Although the relative abundance of the individual in-gel-stained bands indicated a highly dynamic fluctuation during the recovery period (Figure S4), the highest proteolytic activity was registered after 7 d of recovery both under RAF and RAD. 

### 2.6. Contribution of Stress-Induced Proteins for the Recovery of Desiccated Plants

#### 2.6.1. Dehydrins

**Figure 9:**
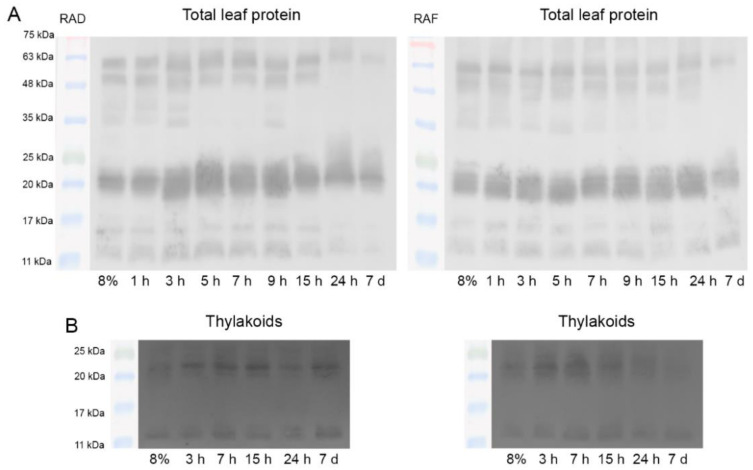
Changes in the relative amount of LEA2 group stress-induced proteins, detected by Western blot analysis against conserved K-segments of dehydrins in total leaf proteins (**A**) and in thylakoids (**B**) isolated from *H. rhodopensis* plants during the recovery from drought- (RAD) and freezing-induced desiccation (RAF).

The protein pattern of dehydrins, LEA2 group stress-induced proteins, during RAD and RAF was investigated by Western blot (Figure 9). Immunoblots of total leaf protein samples showed the presence of several bands with molecular weights between 65 and 12 kDa (65, 60, 50, 45, 38, 20–22, 15, and 12 kDa). The band around 20–22 kDa consisted of two or three different bands. The protein pattern of expressed dehydrins during RAD and RAF was almost identical with a small difference at 45 kDa, where an additional dehydrin appeared only under RAD. The content of stress-induced dehydrins remained high up to 9 and 24 h under RAD and RAF, respectively. After that, their contents began to decline. Rehydration-induced increase of dehydrins (around 22 kDa) was characteristic for RAD from 3 up to 9 h of recovery.

Immunoblot signal from thylakoid samples showed that low molecular-weight dehydrins (20–22 and 12 kDa) were present in the thylakoid membranes under both RAD and RAF, and the bands were more pronounced under RAF. During RAD, the content of dehydrins started to increase after 3 h of recovery and remained at this level until 7 d of rehydration (Figure 9B).

#### 2.6.2. ELIPs

**Figure 10:**
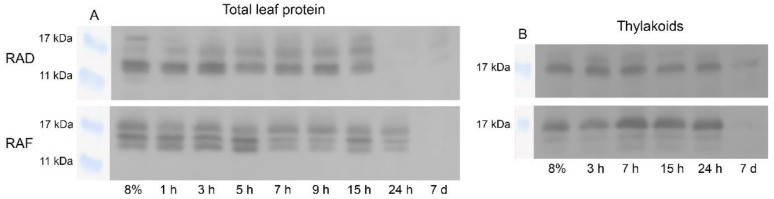
Changes in the relative amount of ELIPs, detected by Western blot analysis with anti-ELIP antibody in the total leaf proteins (**A**) and in thylakoids (**B**) isolated from *H. rhodopensis* plants during rehydration after drought- (RAD) and freezing-induced (RAF) desiccation.

The protein abundance and pattern of the ELIPs was monitored by Western blot during the early hours of RAD and RAF. We identified three main bands in isolated total leaf protein samples differing in molecular weight (14–18 kDa) that showed distinct abundance patterns during RAD and RAF (Figure 10). ELIP proteins were present in all samples up to 15 and 24 h under RAD and RAF, respectively, whereas at 7 d of rehydration, only faint ELIP bands were detected. Immunoblot signals from isolated thylakoid membranes showed the presence of two or three main bands during RAD and RAF, respectively, with apparent molecular weights of 14–18 kDa (Figure 10B). The relative protein abundance of these bands altered during rehydration. In general, high ELIP contents were detected in the investigated samples, except in those after 7 d rehydration. A relatively high ELIP signal was detected in isolated thylakoids at 24 h of RAD, whereas the ELIP signal was weak for the total leaf proteins, which indicates a selective removal of ELIPs during rehydration, and they also remained in the thylakoids in the later stages of the recovery. The presence of the ELIP signal in the total protein after 24 h of RAF suggests that RAF is a more complex process in which ELIP protection is required for a longer period. These results are in accordance with the time scale of the rehydration-induced decrease of the fluidity of the thylakoid–lipid matrix. 

### 2.7. Relative Transcript Amount of ELIP Genes

The presence of several protein bands cross reacting with the antibody raised against pea (*Pisum sativum*) ELIP protein prompted us to examine the relative transcript abundance of their closest *H. rhodopensis* homologs. A local TBLASN search against the published *H. rhodopensis* RNAseq contigs database [41] using the ELI_PEA (Acc. No SP:P11432) protein sequence as a query identified four contigs encoding proteins with high similarity. For the sake of clarity, we named those *ELIP1* to *ELIP4*, where *ELIP1* showed the highest similarity and *ELIP4* the lowest (Data S1 and Figure S5). Although it did not show high homology to the pea ELIP protein, in the present study we also included *NA2*_*ELIP*, as its expression has been previously examined [18], and therefore validated primers were readily available. According to clustering based on RNAseq transcript counts in the course of desiccation and rehydration, the expression patterns of *NA2_ELIP* and *ELIP4* were most similar, and *ELIP1* and *ELIP3* were also clustered together, while *ELIP2*, the one with lowest transcript counts, showed a different pattern (Figure S6).

**Figure 11:**
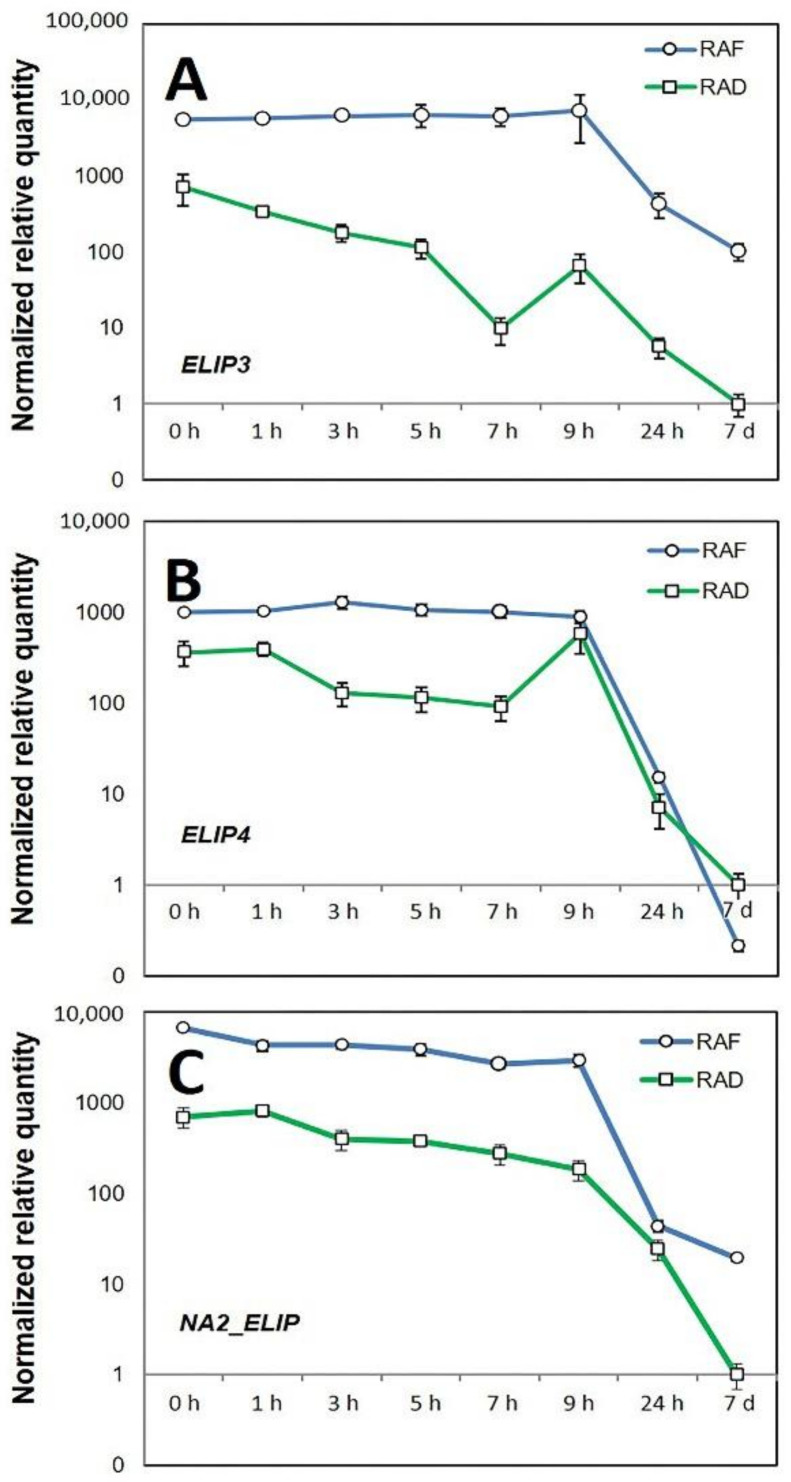
Relative transcript abundance of the *ELIP* genes: *ELIP3*: Contig_024549 (**A**); *ELIP4*: Contig_093552 (**B**); and *NA2-ELIP*: Contig_093441 (**C**) during recovery of *H. rhodopensis* after drought- (RAD) and freezing-induced desiccation (RAF). Normalized relative transcript abundances were scaled to the expression measured at 7 d of RAD, and the corresponding standard deviations are given (*n* = 3).

Our attempts to design specific qRT-PCR primers for *ELIP1* and *ELIP2* encoding contigs with three different pairs of primers for each contig were not successful. Therefore, the expression of the remaining three RNAseq contigs encoding ELIP proteins were studied by qRT-PCR (Table S1). The overall expression patterns of the three studied genes were similar, showing high expression levels in the desiccated state and then declining at different paces during recovery. The transcript abundance of the studied *ELIP* encoding contigs at the desiccated state was 366–6653-fold higher, compared to the steady-state levels in fully recovered condition after 7 d under RAD. These high levels were maintained for up to 9 h of recovery and declined to nearly unstressed level at 24 h (Figure 11). However, both the expression levels and patterns of each *ELIP* gene were distinct under RAD and RAF. All studied genes showed higher expression during RAF compared to RAD. The highest difference between RAF and RAD (nearly 634-fold) was observed in the relative transcript abundance of *ELIP3* (Contig_024549) at 7 h. Although the relative expression of *ELIP4* (Contig_093552) during both RAF and RAD was lower than that of *NA2-ELIP* (Contig_093441), both genes maintained around 10-fold higher expression between 3 and 7 h of RAF. The pace of expression decline was also different for each gene. In contrast to RAD, during which *ELIP3* (Contig_024549) expression slowly declined up to 7 h then peaked again at 9 h followed by a sharp decline to 7 d, its expression during RAF was maintained higher and almost unchanged up to 9 h. However, the decline during RAF was somewhat slower, reaching expression levels nearly 100-fold higher than after 7 d of RAD. Similarly, slower expression decline during RAF was observed for *NA2-ELIP*, but its levels at 7 d were 19-fold higher compared to RAD. In contrast, its expression decline during RAF appeared to be sharper than during RAD for *ELIP4* where the transcript abundance at 7 d dropped below that observed for RAD. *ELIP4* also showed a distinct expression pattern during RAD, with its expression significantly dropping between 1 and 3 h, staying at this level up to 7 h, and then peaking at 9 h (Figure 11B). These data provide evidence for a coordinated expression of *ELIP* genes during recovery and suggest a distinct role for each ELIP protein in protecting the photosystem during RAD and RAF.

## 3. Discussion

Recovery of the HDT plant *H. rhodopensis* is a unique process that restores physiological activity after drought-induced desiccation or after freezing- induced desiccation [6]. Nevertheless, it is important to note that the leaves of plants desiccated by freezing stress were exposed to the combined effects of cellular-level drought and low-temperature stresses. In fact, they were subjected to subzero temperatures in a desiccated state for more than one month and remained in a dry state for more than 2 months before rehydration, as was presented by Mihailova et al. [6].

Upon watering, rehydration was found to be a slow process during the first 15 h both under RAF and RAD. Gradual water uptake resulted in limited oxidative stress, and prevention against cellular damage seems to be critical for later survival, as rapid plant rehydration has been shown to trigger the additional accumulation of hydrogen peroxide and induce adverse changes in the activity of the photosynthetic apparatus [42]. 

### 3.1. Recovery of the Ultrastructure of Cells and Chloroplasts

Ultrastructural changes (including the disappearance of secondary vacuoles and the concomitant return of the chloroplasts to the cell periphery) observed both under RAD and RAF seem to be a reversion of processes observed during desiccation [28]. It should be mentioned that chloroplasts remained intact in the desiccated stage, and they gradually assumed the shape and thylakoid distribution typical of control tissue without apparent damage. It is important to note that under both RAD and RAF, structural alterations proceeded similarly in sequence, but with a phase shift, resulting in RAD lasting longer compared to RAF (Figure 1, Figure 2, Figure 3 and Figure 4). This shows that *H. rhodopensis* can avoid frost damage by undergoing desiccation, to which it has accommodated earlier. On the other hand, results on membrane fluidity and the removal of ELIP proteins underline that not all the processes align the faster recovery, indicating that preliminary frost stress alters the sequence of recovery processes.

In order to monitor the structural alterations in the thylakoids, we evaluated granum RD values, which proved to be effective parameters for monitoring slight changes in thylakoid structure related to different abiotic stresses [43,44]. Based on previous works [6,28], the granum structure of samples desiccated in a drought- or freezing-induced manner was fully retained in the desiccated state (Table 1), which underlines the effectiveness of mechanisms protecting thylakoid membrane complexes and lipids. Similarly, RD values remained unchanged under RAF. Drought-induced desiccation resulted in a shrinkage and thus decrease in RD, which was fully restored after rehydration (Table 1). This might indicate different water-retaining capacity or granum structural stabilization under freezing- and drought-induced desiccation. Indeed, restoration of RD under RAD indicated that protective mechanisms are sufficient enough to avoid severe damage during the desiccation. 

### 3.2. Changes in the Thylakoid Lipid Component

Although the structure of thylakoids found to be resistant against desiccation and their subsequent recovery, we found slight alterations in the fluidity of the thylakoid membranes, the restoration of which was gradual both under RAD and RAF. The optimal fluidity of thylakoid membranes allows for the rearrangement and translocation of pigment–protein complexes in order to preform effective photosynthetic process and to facilitate protection when plants are exposed to unfavorable environmental stress factors [45,46]. The susceptibility of plants to chilling has been related to a particular composition of the lipid phases for retaining an optimal fluidity, which is of primary importance for plant tolerance to low temperatures [47]. The lipid composition of the thylakoid membranes of higher plants is unique among biological membranes, containing 80–90% galactolipids—monogalactosyldiacylglycerol (MGDG) and digalactosyldiacylglycerol (DGDG)—which are characterized by a high degree of unsaturation [45,48], represented by 18:3 and 16:3 fatty acids that comprise nearly 70% of total fatty acid content [48]. Our results indicate that thylakoids of *H. rhodopensis* showed very high fluidity during the lipid phase as a result of water deprivation from drought or freezing (RWC around 8%) which can be attributed to the increased population of polyunsaturated fatty acids in membrane lipids providing fluid environment under these extreme conditions of desiccation [49]. In the freezing-induced desiccation stage, the fluidity of the thylakoid membranes was even higher compared to plants that had been desiccated by drought. This difference reflects on the accelerated synthesis of unsaturated fatty acids upon low-temperature stress, which increased their tolerance to chilling stress [47,50]. After 15 h of RAD and RAF, the fluidity of the lipid phase of thylakoid membranes of *H. rhodopensis* slowly started to decrease, and after 7 d, the fluidity of the lipid phase, estimated by fluorescence polarization of DPH, reached values of about 0.25–0.28, which are characteristic for plant membranes and were also found in *Pisum sativum* [51] and *Arabidopsis thaliana* [52]. It has been reported that during the dehydration and rehydration of HDT plant *C. pumilum*, the alterations of the thylakoid membranes’ protein content and the rearrangement of complexes were accompanied by changes of lipid phase properties—a formation of H_II_ phases (inverted hexagonal micelles) was observed [17]. Our data showed that maintenance of the photosynthetic competence and the ability of recovery from desiccation are related and accompanied with alteration of the lipid phase properties. 

### 3.3. Changes in Thylakoid Complexes and Photosynthetic Proteins

The modifications in thylakoid organization demonstrated by the shift in the ratios of the main pigment–protein complexes and changes in their assembly forms showed no significant differences between RAD and RAF. In the desiccated stage, elevated PSI/PSII and LHCII/PSII ratios are characteristic due to a more substantial decline in PSII content compared to those of the other complexes [53]. Desiccation induced substantial and moderate decreases in PSI-LHCII and PSII-s, respectively, and increased Lhc-m values correspond to the slight disassembly of the photosynthetic machinery. The larger decrease in PSII-s as a result of freezing-induced dehydration compared to drought-induced desiccation [6] corresponds to the inactivation of PSII reaction centers that is also typical for evergreen temperate plants [54]. During rehydration, reorganization of thylakoid complexes started in the direction of the structure of well-hydrated plants, also recorded in previous studies [6,53]. The reorganization process seemed to be light dependent, since at the 15 h and 24 h time points, which were recorded after the night period, the trend of compositional alterations was found to be less intensive compared to 7 h of recovery. The most obvious compositional change, identical under RAD and RAF, was the increase in the proportion of the PSI-LHCII complex (Figure 6A), which corresponded to the reduced F_678_/F_650_ ratio, indicating an increase in the involvement of Chl *b* in the energy supply of PSI, as we reported earlier [40]. In addition, with the onset of rehydration, a decrease in the amount of Lhc-m started, and the proportion of PSII-s complexes gradually increased up to 7 d. The pattern of increase in PSII-s amount was more-or-less in line with PSII reactivation [40].

The rather unchanged PSI/PSII ratio (Figure 5A) was supported by immunoblot data both under RAD (Table 2) and RAF (Table 3). In spite of the unchanged ratio, the amount of D1 protein gradually increased during RAD and RAF, whereas that of D2 remained rather stable under RAD and only changed significantly under RAF. Similar to the unchanged PSI/PSII ratio, the level of LHCI and LHCII apoproteins (Lhca-s and Lhcb-s), with a slight increase in Lhca1 and Lhcb2 content, also proved to be rather stable during RAD and RAF. In consequence, photosynthetic proteins were largely maintained in the desiccated stage [14,16,55], thus neither recovery processes led to a significant alteration of this status. However, increased abundance of PsbS during both RAD and RAF supports its role in increased thermal energy dissipation, which was previously described during rehydration [40]. The maintenance of high levels of other proteins, such as Lhcb6 and Lhcb5, during rehydration probably also contributes to the increased thermal energy dissipation. 

Compared to chlorophyll–protein complexes, compositional changes to the non-chlorophyll–protein complex members of the photosynthetic electron transport chain as well as protective proteins of the photosynthetic apparatus are even less characteristic during the rehydration of HDT plants, although the importance of the cyt *b_6_f* complex in tolerance mechanisms under drought stress was suggested. Charuvi et al. [17] observed a substantial reduction in the level of cyt *f* during early dehydration of *C. pumilum* and proposed that the initial regulation of the inhibition of electron transport is achieved via the cyt *b_6_f* complex. In addition, drought stress-induced decrease in the content of the Rieske iron–sulfur protein of the cyt *b_6_f* complex (PetC) is considered as the key factor determining full recovery of photosynthetic apparatus [42]. It was proposed that the unchanged level of PetC protein under drought stress and rehydration is a requisite for full plant recovery after stress. Our results clearly showed increased abundance of all the proteins of the cyt *b_6_f* complex during rehydration, especially under RAD (Table 2 and Table 3). It could be suggested that elevated levels of cyt *b_6_f* complexes are associated with increased cyclic electron flow during both RAD and RAF [56].

Oxidative damage supposed to occur during rehydration could lead to the decline in the amounts of certain proteins. In addition, even a 7 d period was not long enough to achieve complete recovery in the levels of multiple photosynthetic proteins. Incomplete recovery of RbcL, PSI reaction center proteins, PsbO, and PsbC has also been reported for resurrection bryophytes *Selaginella bryopteris* and *Fontinalis antipyretica* and the HDT angiosperm *H. rhodopensis* [16,57,58]. This incomplete recovery seems to be responsible for the incomplete restoration of CO_2_ fixation and O_2_ evolution that was also reported by earlier studies [6,15,57,59]. 

### 3.4. Proteins with Stress Defensive Function during Rehydration

Among defensive proteins that could protect thylakoid complexes, the differences in the protein pattern of ELIPs under RAD and RAF (Figure 10) indicate a variation in the protection that maintains the thylakoid complexes. Although the induction of ELIPs was mainly reported for the desiccation stage and their important role against photooxidative damage has been demonstrated so far [19,60], our results suggest their role in the initial phase of recovery. ELIPs have been extensively studied in land plants as target genes to enhance tolerance against high light stress [61]. VanBuren et al. [62] reported a massive tandem reduplication of *ELIP* genes in resurrection plants, showing that resurrection plants encode, on average, 20.7 *ELIP* genes per genome, while only 3.1 *ELIP* genes are encoded in the sensitive species. In consequence, ELIPs seem to have an important role in forming vegetative desiccation tolerance. In multiple resurrection plants ELIPs are among the most abundant transcripts during dehydration and rehydration [63,64], with increasing transcript abundance throughout dehydration reaching the highest amount in desiccated tissues. This high relative transcript amount of ELIPs is generally maintained during early rehydration and then decreases to the level of unstressed well-hydrated plants [62]. Our results confirm this pattern in *H. rhodopensis* and are in agreement with RNAseq data [41]. 

However, we first compared the expression of *H. rhodopensis* ELIPs during RAD and RAF at both the protein and mRNA levels. The high *ELIP* transcript and protein abundances under both drought- and freezing-induced desiccated states suggests that ELIP proteins, as well as their mRNAs, are being stored during desiccation, and are thus immediately available once needed for chloroplast regeneration [65]. Our data also support the existence of this mechanism in *H. rhodopensis.* The observed up to 10-fold higher expression of the studied *ELIP* genes in the early stages of RAF, compared to RAD, implies that freezing stress might cause more photooxidative damage, and thus more ELIPs are required to mitigate its effect. In *Chlamydomonas reinhardtii* the expression of *ELIP* can enhance the resistance to cold-induced photooxidative stress [61]. The distinct expression patterns of the three studied *ELIP* genes suggest that the expression of individual *ELIP* genes is coordinated depending on the specific conditions, and each gene may play a different role during RAF and RAD. The observed different pace of the return of the expression of *ELIPs* to fully hydrated levels may also have a physiological role. However, further studies are needed to elucidate the exact mechanisms of their regulation and action.

Similar to ELIPs, dehydrins also proved to be important in the initial hours of recovery both under RAD and RAF. In fact, the content of dehydrins remains high up to 9 and 24 h during RAD and RAF, respectively (Figure 9). The low molecular weight of dehydrins (20–22 and 12 kDa) presented in the thylakoid membranes was more pronounced under RAF. Regardless of the significant reduction of dehydrin contents after 7 d of rehydration, some of them are still present. Expression analysis of dehydrin isoforms during dehydration and subsequent rehydration in the leaves, roots, and callus of the resurrection species *C. plantagineum* revealed that dehydrin transcripts were still existent in rehydrated samples [66]. Layton et al. [37] showed that a 31-kDa dehydrin was detected during the drying of resurrection fern *Polypodium polyploidies* but disappeared 24 h after rehydration. Surprisingly, similar amounts of dehydrins were quantified in control, desiccated, and 24 h-rehydrated lichenic algae *Trebouxia erici* [49]. It has been suggested that in poikilohydric organisms, LEA proteins play a role in protecting cellular constituents during both drying and rehydration [67].

### 3.5. Proteolysis Controls Protein Turnover under Recovery

Reorganization processes in the leaf cells require the decomposition of proteins that are not further necessary, especially for the protection of cell constituents. In our measurements, the highest proteolytic activity was registered at 7 d both under RAF and RAD (Figure 8 and Figure S4). In order to eliminate irreversibly damaged proteins as well as to provide monomers for de novo protein synthesis, proteases are involved in the stress response, especially under prolonged and/or severe conditions [68]. The presence of multiple proteases has been reported in desiccated leaves of *Ramonda serbica* [69]. Considerably less is known about the role of proteases in rehydration. A surprising finding in our study was the conserved protease activity in leaves under freezing-induced desiccation compared to drought-induced desiccation, which had low activity. Increased proteolysis could contribute to cold acclimation [70,71], whereas low proteolytic activity has been also detected in drought-resistant wheat varieties under prolonged stress contrary to the more sensitive ones [72,73]. Upregulation of proteases has been reported in the resurrection fern-ally *Selaginella tamariscina* after 12 h of rewatering following desiccation [74]. In our study the dynamic changes in protease activities in the time course of recovery could be related to dynamic changes in the protein content, which is reported to be higher in the desiccated resurrection plant *R. serbica* than in the rehydrated ones [69].

## 4. Materials and Methods

### 4.1. Desiccation and Rehydration of Plants

*Haberlea rhodopensis* Friv. tufts of the shade ecotype were initially collected from the Rhodope Mountains and further cultivated under ex situ environmental conditions. Desiccation and rehydration of plants were performed as described by Georgieva et al. [40]. To study the recovery from drought-induced desiccation (RAD), *H. rhodopensis* plants were desiccated to an air-dry state in a climatic chamber, FytoScope FS 130 (Photon Systems Instruments, Drásov, Czech Republic) at 25/18 °C day/night temperatures, 60% relative humidity, 12 h photoperiod, and a photosynthetically active photon flux density (PPFD) of 25 µmol photons m^−2^ s^−1^. To study recovery from freezing-induced desiccation (RAF), tufts were kept under environmental conditions continuously (average daily maximum PPFD of 30–60 µmol photons m^−2^ s^−1^), where they were exposed to natural cold and freezing temperatures during autumn and winter. When the temperature dropped to about –10 °C, the dehydration of the plants began, and they overwintered in an air-dry state. The rehydration of plants after drought- and freezing-induced desiccation was carried out in laboratory conditions at 21–23 °C and a PPFD of 25–30 µmol photons m^−2^ s^−1^. Initially, the soil substrate was well watered, and then the pots were placed in a modified desiccator, maintaining a high constant humidity via a water pump. Measurements were conducted on dry leaves (0 h) and after 1, 3, 5, 7, 9, 15, 24 h, and 7 d of rehydration. Relative water content (RWC) was determined as described previously [14].

### 4.2. Transmission Electron Microscopy (TEM)

Leaf pieces from the central leaf blade region of rehydrated rosettes were fixed in 2.5% glutaraldehyde (70 mM K-Na phosphate buffer, pH 7.2) for 2 h at room temperature, then post-fixed in 1% OsO_4_ in the same buffer for 1.5 h. Samples were dehydrated in a graded ethanol series, incubated in propylene oxide, and embedded in Durcupan ACM (Fluka). Ultrathin (70 nm) sections were made with Ultracut E (Reichert-Jung, Vienna, Austria) ultramicrotome, stained with uranyl acetate and lead-citrate, and examined with a JEM-1011 (JEOL, Tokyo, Japan) electron microscope equipped with a Morada digital camera (Olympus, Tokyo, Japan) and ITEM software (Olympus, Tokyo, Japan). Measurements of the chloroplast dimensions as well as fast Fourier transformation (FFT) on the selected regions of interest of particular micrographs were performed using ImageJ (NIH, US) software in order to determine the granum repeat distance (RD) values according to Ünnep et al. [43].

### 4.3. Steady-State Fluorescence Polarization Measurements

For the estimation of alterations to the thylakoid membrane fluidity during the rehydration of *H. rhodopensis*, the method for measuring of the degree of polarization of the steady state fluorescence emitted from the 1,6-diphenyl-1,3,5-hexatriene (DPH) probe at room temperature, as described previously [51], was applied. DPH tends to distribute evenly between all lipid domains, and as no energy transfer occurs between DPH and photosynthetic pigments, it is very suitable for determining the fluidity of the hydrophobic interior of biological membranes and especially of thylakoid membranes. DPH was added to thylakoid membranes to a final concentration of 2.5 µM from a stock solution in tetrahydrofuran. Measurements were performed at room temperature in the resuspension buffer (0.33 M sucrose, 5 mM MgCl_2_, 10 mM NaCl, and 20 mM Tricine, pH 7.5) using a JASCO FP8300 fluorometer (Jasco, Tokyo, Japan), equipped with polarization filters. Fluorescence was excited at 360 nm and registered at 460 nm, and the chlorophyll concentration in samples was 11 µg mL^−1^. The degree of polarization (P) was estimated using a formula described previously [51].

### 4.4. Thylakoid Isolation and 2D Blue Native/SDS PAGE

*H. rhodopensis* thylakoids were isolated according to Georgieva et al. [14]. Osmotic shock, removal of coupling factor (CF_1_), determination of the chlorophyll content [75], storage, and separation of complexes were carried out as described previously [76]. Briefly, complexes were separated in 4.3–12% Blue Native (BN) gel gradient. Prior to loading, thylakoids were washed in 50 mM BisTris-HCl, pH 7.0, 330 mM sorbitol, 250 µg mL^−1^ Pefabloc, and pelleted by 10 min centrifugation with 10,000× *g*. Washed thylakoids were solubilized in 750 mM aminocaproic acid, 50 mM Bis-Tris, pH 7.0, 0.5 mM EDTA with 1% (*w*/*V*) *n*-dodecyl-*β*-D-maltoside (*β*-DM, Sigma, Saint Louis, MO, USA) plus 1% (*w*/*V*) digitonin (Serva, Heidelberg, Germany) at 500 µg chlorophyll mL^−1^ concentration followed by 15 min centrifugation with 18,000× *g*. The polypeptide patterns of complexes were obtained by applying cut BN lanes on the top of 10–18% SDS gel gradient containing 8.7% (*w*/*V*) glycerol. After running, gels were stained with the Blue–Silver method [77]. For gel electrophoreses, the Mini-Protean apparatus (BioRad, Hercules, CA, USA) was used. BN and SDS PAGE were carried out, and gel patterns were evaluated according to Sárvári et al. [76]. The gels were scanned using an Epson Perfection V750 PRO scanner. Densitometry analysis of gels was carried out using the Phoretix image analysis software (Phoretix International, Newcastle-upon-Tyne, UK).

### 4.5. Thylakoid and Total Leaf Protein Isolation, SDS-PAGE and Western Blot

Thylakoid membranes were isolated according to Georgieva et al. [14], and isolated thylakoid samples were solubilized in the sample buffer (50 mM Tris-HCl, pH 6.8, 2% (*w*/*V*) SDS, 2% (*V*/*V*) *β*-mercaptoethanol and 10% (*V*/*V*) glycerol). Total leaf proteins were extracted in sample buffer as described by Mihailova et al. [6]. Samples were separated by SDS-PAGE (SE260 Mighty Small II, Hoefer, USA) according to Laemmli [78], modified by adding 8.0% (*V*/*V*) glycerol to stacking and separating gels using a constant current of 20 mA per gel. Thylakoid samples corresponding to 2 µg chlorophyll (20 µg protein) or 30 µg total leaf protein were applied per lane. Semi-dry transfer (TE70X, Hoefer, Holliston, MA, USA) was used to blot the proteins on nitrocellulose membrane (90 min at a current of 1 mA cm^−2^). ROTI^®^Mark TRICOLOR (Carl Roth GmbH + Co. KG, Karlsruhe, Germany) was used as a pre-stained protein standard for monitoring electrophoretic separation and transfer efficiency. Blots were probed with primary antibodies against PsaA (AS06 172), PsaB (AS10 695), PsbA (AS05 084), PsbD (AS06 146), PsbC (AS11 1787), PsbB (AS04 038), PsbO (AS06 142-33), PsbQ (AS06 142-16), PsbS (AS09 533), PetA (AS06 119), PetB (AS18 4169), PetC (AS08 330), Lhcb1 (AS01 004), Lhcb2 (AS01 003), Lhcb3 (AS01 002), Lhcb4 (AS04 045), Lhcb5 (AS01 009), Lhcb6 (AS01 010), Lhca1 (AS01 005), Lhca2 (AS01 006), Lhca3 (AS01 007), Lhca4 (AS01 008), ELIP (AS06 147A), and dehydrin-conserved K-segment (AS07 206A) (Agrisera, Vännäs, Sweden). Horseradish peroxidase-conjugated goat anti-rabbit secondary antibody was used (AS09 602, Agrisera, Vännäs, Sweden). The resulting bands were visualized by enhanced chemiluminescence, and signals were recorded on X-ray Blue films (Carestream Dental LLC, Atlanta, GA, USA). Films were scanned using an Epson Perfection V850 PRO scanner, and densitometry was made by Gel-Pro Analyzer software (Media Cybernetic, Rockville, MD, USA).

### 4.6. Protease Activity

For protease activity estimation, 250–500 mg leaf material was ground in liquid nitrogen. Proteins were extracted as previously described [79] in an ice-cold medium containing 50 mM potassium phosphate buffer (PPB, pH 7.8), 10 mM KCl, 1 mM EDTA, 1.25 mM PEG 4000, 0.5 M sucrose, 20 mM ascorbic acid, 10 mM dithiothreitol (DTT), 0.1% (*V*/*V*) Triton X-100, and 2% (*w*/*V*) Polyclar AT, and centrifuged at 15,000× *g* for 30 min at 4 °C. Total soluble protein content was determined according to Bradford [80] at 595 nm using bovine serum albumin as a standard. Proteins mixed with sample buffer without boiling were separated at 8 °C by SDS-PAGE with 10% resolving gel containing co-polymerized 0.05–0.1% (*w*/*V*) gelatin as a substrate, and 5% concentrating gel in a Mini Protean II Dual Slab Cell (Bio-Rad). After electrophoresis, SDS was chased from the gels and proteases activated by incubation with 2% (*V*/*V*) Triton-X-100 for 60 min at room temperature (two changes of the detergent, the second with 5 mM cysteine in addition, continuous shaking). Next, gels were incubated for 22 h at room temperature in 100 mM potassium phosphate buffer, pH 6.0, containing 5 mM cysteine, or in 100 mM Tris-HCl buffer supplemented with 2 mM CaCl_2_ and 5 mM cysteine for developing the enzyme activity, then stained with colloidal Coomassie dye. Protease activity was revealed as white bands on a blue background. Gel images were taken using the UVItec gel documentation system (Cambridge, UK) and analyzed using Gel-Pro32 Analyzer software (Media Cybernetics, Rockville, MD, USA). The protease activity of each separated band corresponded to its peak intensity, which was given as the total integrated optical density (IOD) in arbitrary units. Total activity for a particular treatment was considered to be the sum of IOD values of the bands in a lane. Proteolytic activity in desiccated plants was assumed to be 100%, and the values during rehydration were calculated relative to this selected value.

### 4.7. Identification of ELIP Protein Encoding RNAseq Contigs and qRT-PCR Primers Design

To identify *H. rhodopensis* contigs encoding homologous proteins, the protein sequence of *Pisum sativum* Early Light-Induced Protein (Acc. No SP: P11432) was used as a query in a local TBLASTN search [81] against the entire database of *H. rhodopensis* RNAseq contigs [41]. The identified contigs, showing highest similarity of the encoded protein, were selected for qPCR analysis. Annotations were updated by running a BLASTX search against the flowering plants (taxID:3398) non-redundant protein database at NCBI (https://blast.ncbi.nlm.nih.gov/Blast.cgi) accessed on 10 June 2022. The qRT-PCR for HrActin (GB: GT270756), *ELIP1* (Contig_003481), *ELIP2* (Contig_093673), *ELIP3* (Contig_024549), and *ELIP4* (Contig_093552) were designed using the Primer3plus web server at https://www.primer3plus.com (accessed on 28 February 2022) by selecting the server default settings for qPCR primers’ design, and the remaining two primers were selected from a previously published paper [18] (primers are listed in Table S1). The designed primers were tested for specificity by the EMBOSS program “primersearch” [82] against *H. rhodopensis* RNAseq contigs [41].

### 4.8. RNA Extraction and qRT-PCR

Total RNA was extracted from 100 mg of leaf tissue, which had been flash frozen in liquid nitrogen after 0 h, 1 h, 3 h, 5 h, 7 h, 9 h, 24 h, and 7 d after the start of watering of RAF and RAD plants. The extraction was done by using the Trizol^®^-based Direct-zol RNA miniprep kit (Zymo Research, Irvine, CA, USA) and was in-column treated with DNAase I according to manufacturer’s instructions. RNA quantity and quality was determined using a NanoDrop 1000 spectrophotometer (Thermo Fisher Scientific, Wilmington, DE, USA) and confirmed by “Bleach gel” electrophoresis [83]. A PrimeScript™ RT reagent Kit with gDNA Eraser, Cat. #RR047A (Takara Bio, Shiga, Japan) and 1 μg total RNA (in column treated with DNAase I) were used for the first strand cDNA synthesis. According to the kit developer instructions, the RNA was treated with the provided gDNA Eraser for 2 min at 42 °C prior to first-strand cDNA synthesis. The reverse transcription reaction was performed at 37 °C for 15 min, using the RT primer mix (containing Oligo dT Primer and Random 6 mers) provided with the kit, followed by deactivation of the RT enzyme for 5 s at 85 °C. 

Quantitative PCR (qPCR) was performed on an ABI 7300 Real-Time PCR System (Thermo Fisher Scientific, Waltham, MA, USA) and was carried out in 20 μL reaction volume in triplicates. The reaction mixes consisted of 1× TB Green Premix Ex Taq (Tli RNase H Plus) Cat. # RR420L (Takara Bio, Shiga, Japan), 2 µM each of forward and reverse primers, 0.4 µL 50x ROX dye, and 2 μL of 20× diluted first-strand cDNA. For all reactions, with the exception of Contig_024549 (*ELIP3*), the cycling conditions were following the two-step protocol recommended by the manufacturer, and included initial denaturation for 30 s at 95 °C, followed by 40 cycles of 95 °C 5 s 62 °C 31 s. The qPCR protocol for Contig_024549 (*ELIP3*) required additional optimization which included lowering primer concentrations to 1 μM each and applying a 3-step amplification protocol for 40 cycles at 95 °C 5 s; 62 °C 15 s; and 72 °C after initial denaturation at 95 °C for 30 s. Data acquisitions were performed with the SDS v1.4 software (Thermo Fisher Scientific, Waltham, MA, USA). For relative quantification, the Actin 7 (HrhActin, HrhDR Haberlea rhodopensis cDNA, mRNA sequence, Acc. No GenBank: GT270756) was selected as reference gene, since its expression was more stable across all studied RAD and RAF samples than that of the previously used Heat shock protein 90 (HSP90, Contig_000452) [31], data not shown). Relative expression changes were calculated by genex, a BioRad MS Excel Gene Expression macro v1.1 (BioRad, Hercules, CA, USA) according to the Pfaffl method [84]. For each gene, the relative expression was scaled to the expression at 7 d of RAD as calibrator.

### 4.9. Statistics

The rehydration of desiccated plants as a result of both drought and freezing stress was repeated twice. At each time point, leaves from 6 different tufts were collected and the mean samples were used for all analyses. Changes in the investigated parameters between RAD and RAF plants were statistically compared by the Fisher least significant difference test at *p* ≤ 0.05 following ANOVA. A statistical software package (Statgraphics Plus, version 5.1 for Windows, The Plains, VA, USA) was used.

GraphPad Prism 8 (GraphPad Software, San Diego, CA, USA) was used for the statistical analyses of data derived from TEM images, such as normality test, ANOVA, and post hoc tests. Since data did not follow normal distribution, the Kruskal–Wallis non-parametric ANOVA test was performed, followed by Dunn’s multiple comparisons test as the post hoc test. Significant differences were labeled with different letters.

## 5. Conclusions

In conclusion, processes that support the recovery from desiccation caused by the environment show slight natural variations. In consequence, the reactivation of the photosynthetic apparatus can follow multiple paths in *H. rhodopensis* under RAD and RAF. The ultrastructure of the cells under RAD recovered more slowly compared to RAF, and shrinkage of thylakoids in air-dried plants was visible only under RAD. The amount of the main photosynthetic proteins increased in the first hours of rehydration under RAD (3–15 h), whereas under RAF their content decreased. Also, higher expression of *ELIPs* was found under RAF compared to RAD. Nevertheless, there are common mechanisms, indeed, that contribute to the fast regain of photosynthetic function in *H. rhodopensis*, regardless of the cause of the desiccation. These are: (1) enhanced fluidity of the lipid phase of thylakoid membranes in the air-dry state and during the first hours of rehydration allows for the rearrangement of pigment–protein complexes during rehydration and faster restoration of PSII and PSI functions; (2) elevated proportion of the PSI-LHCII complexes suggests an increase in the involvement of Chl *b* in the energy supply of PSI; (3) enhanced levels of cyt *b_6_f* complexes that facilitate the acceleration of electron flow under rehydration, thus providing sufficient reducing equivalent for the recovery process; (4) high abundance of proteins related to thermal energy dissipation, such as PsbS, Lhcb5, Lhcb6, and ELIPs; (5) higher proteolytic activity that allows for the elimination of damaged proteins or provides intermediate metabolites and building blocks for de novo synthesis of proteins; and (6) high amounts of dehydrins that protect the subcellular structural integrity. These core mechanisms seem to be crucial for the effectiveness of the restoration of photosynthetic function.

## Figures and Tables

**Table 1:** Chloroplast ultrastructural features of *H. rhodopensis* during the recovery from drought- (RAD) and freezing-induced desiccation (RAF) as measured on TEM micrographs. Statistically significant differences among different samples (*n* is indicated for all groups) are indicated with different letters (*p* ≤ 0.05) within the different columns.

	Chloroplast			Granum
Variants	Length (nm)	Width (nm)	L/W Ratio	Repeat Distance (nm)
RAD 0 h	2844 ± 1271 ^bc^ (*n* = 27)	2117 ± 920 ^b^ (*n* = 27)	1.3 ± 0.2 ^c^ (*n* = 27)	17.7 ± 2.3 ^b^ (*n* = 70)
RAD 24 h	2857 ± 1737 ^c^ (*n* = 69)	2393 ± 1332 ^b^ (*n* = 69)	1.2 ± 0.2 ^a^ (*n* = 69)	24.1 ± 2.4 ^c^ (*n* = 144)
RAF 0 h	3838 ± 770 ^ab^ (*n* = 19)	3390 ± 600 ^a^ (*n* = 19)	1.1 ± 0.1 ^a^ (*n* = 19)	21.8 ± 2.6 ^a^ (*n* = 111)
RAF 24 h	4673 ± 1121 ^a^ (*n* = 30)	1875 ± 479 ^b^ (*n* = 30)	2.6 ± 0.8 ^b^ (*n* = 30)	20.9 ± 1.3 ^a^ (*n* = 104)

**Table 2:** Changes in the main photosynthetic proteins of *H. rhodopensis* plants during rehydration after drought-induced desiccation (RAD). 8%—dried plant; 3, 7, 15 and 24 h—hours after rehydration; 7 d—7 days after rehydration. The abundances of proteins are presented in percentage of the dried plants’ values (8% RWC). Values are given as mean ± SE. The same letters within a graph indicate no significant differences assessed by the Fisher LSD test (*p* ≤ 0.05) after performing ANOVA. The changes in the content of the respective protein between RAD and RAF (Table 3) were statistically compared.

Protein	Abundance (%)					
	8%	3 h	7 h	15 h	24 h	7 d
PsaA	100 ^e^	82 ± 6 ^d^	137 ± 9 ^f^	65 ± 1 ^c^	61 ± 4 ^bc^	84 ± 1 ^d^
PsaB	100 ^d^	117 ± 8 ^ef^	129 ± 6 ^f^	80 ± 6 ^b^	97 ± 7 ^cd^	111 ± 5 ^de^
Lhca1	100 ^a^	115 ± 3 ^b^	122 ± 3 ^cde^	116 ± 4 ^bc^	117 ± 4 ^bcd^	127 ± 3 ^ef^
Lhca2	100 ^c^	95 ± 4 ^bc^	101 ± 3 ^c^	100 ± 2 ^c^	95 ± 3 ^bc^	91 ± 4 ^b^
Lhca3	100 ^c^	121 ± 3 ^de^	134 ± 2 ^f^	93 ± 4 ^bc^	81 ± 3 ^a^	127 ± 5 ^ef^
Lhca4	100 ^cd^	110 ± 2 ^e^	106 ± 3 ^de^	94 ± 2 ^bc^	77 ± 2 ^a^	94 ± 3 ^bc^
PetA (cyt *f*)	100 ^b^	156 ± 6 ^e^	167 ± 15 ^e^	203 ± 11 ^f^	148 ± 9 ^de^	126 ± 8 ^cd^
PetB (cyt b_6_)	100 ^cd^	122 ± 4 ^f^	115 ± 6 ^ef^	137 ± 9 ^g^	104 ± 4 ^de^	89 ± 4 ^bc^
PetC (Rieske)	100 ^b^	125 ± 4 ^c^	158 ± 8 ^d^	177 ± 5 ^e^	95 ± 6 ^ab^	104 ± 5 ^b^
PsbA (D1)	100 ^ab^	126 ± 6 ^bcd^	97 ± 12 ^a^	108 ± 6 ^ab^	121 ± 5 ^abcd^	160 ± 15 ^e^
PsbD (D2)	100 ^de^	97 ± 5 ^de^	92 ± 5 ^cde^	105 ± 5 ^e^	100 ± 6 ^de^	81 ± 8 ^bc^
PsbC	100 ^e^	96 ± 5 ^e^	163 ± 5 ^f^	98 ± 6 ^e^	83 ± 5 ^d^	58 ± 3 ^c^
PsbB	100 ^f^	119 ± 10 ^g^	200 ± 3 ^h^	103 ± 5 ^fg^	76 ± 7 ^de^	84 ± 12 ^e^
Lhcb1	100 ^e^	70 ± 4 ^ab^	94 ± 5 ^de^	60 ± 4 ^a^	96 ± 8 ^de^	62 ± 6 ^a^
Lhcb2	100 ^a^	147 ± 9 ^e^	131 ± 10 ^de^	164 ± 7 ^f^	117 ± 7 ^bcd^	132 ± 5 ^de^
Lhcb3	100 ^d^	84 ± 3 ^ab^	84 ± 4 ^ab^	90 ± 3 ^bc^	91 ± 3 ^bc^	95 ± 3 ^cd^
Lhcb4	100 ^cd^	104 ± 4 ^d^	80 ± 2 ^a^	94 ± 3 ^bc^	83 ± 3 ^a^	92 ± 4 ^b^
Lhcb5	100 ^f^	94 ± 1 ^f^	80 ± 6 ^de^	46 ± 5 ^a^	50 ± 3 ^a^	76 ± 6 ^de^
Lhcb6	100 ^e^	119 ± 0 ^fg^	124 ± 1 ^g^	107 ± 1 ^e^	116 ± 1 ^f^	81 ± 2 ^cd^
PsbO	100 ^ef^	91 ± 5 ^def^	76 ± 5 ^ab^	90 ± 6 ^cde^	90 ± 4 ^cde^	103 ± 3 ^f^
PsbQ	100 ^f^	110 ± 2 ^g^	114 ± 3 ^g^	76 ± 1 ^c^	86 ± 2 ^e^	31 ± 1 ^a^
PsbS	100 ^a^	184 ± 6 ^e^	141 ± 5 ^c^	167 ± 10 ^d^	120 ± 4 ^b^	99 ± 2 ^a^

**Table 3:** Changes in the main photosynthetic proteins of *H. rhodopensis* plants during rehydration after freezing-induced desiccation (RAF). 8%—dried plant; 3, 7, 15 and 24 h—hours after rehydration; 7 d—7 days after rehydration. The abundances of proteins are presented in percentage of the dried plants’ values (8% RWC). Values are given as mean ± SE. The same letters within a graph indicate no significant differences assessed by the Fisher LSD test (*p* ≤ 0.05) after performing ANOVA. The changes in the content of the respective protein between RAD (Table 2) and RAF were statistically compared.

Protein	Abundance (%)					
	8%	3 h	7 h	15 h	24 h	7 d
PsaA	100 ^e^	76 ± 3 ^d^	38 ± 1 ^a^	39 ± 3 ^a^	53 ± 4 ^b^	53 ± 1 ^b^
PsaB	100 ^d^	84 ± 6 ^bc^	57 ± 4 ^a^	52 ± 5 ^a^	73 ± 6 ^b^	73 ± 6 ^b^
Lhca1	100 ^a^	122 ± 3 ^cde^	123 ± 2 ^de^	132 ± 3 ^f^	142 ± 3 ^g^	122 ± 2 ^cde^
Lhca2	100 ^c^	127 ± 5 ^d^	90 ± 2 ^b^	88 ± 4 ^b^	76 ± 3 ^a^	94 ± 5 ^bc^
Lhca3	100 ^c^	115 ± 2 ^d^	87 ± 3 ^ab^	98 ± 4 ^c^	135 ± 5 ^f^	114 ± 3 ^d^
Lhca4	100 ^cd^	110 ± 2 ^e^	77 ± 3 ^a^	76 ± 2 ^a^	91 ± 1.9 ^b^	94 ± 3 ^b^
PetA (cyt *f*)	100 ^b^	118 ± 6 ^bc^	100 ± 8 ^b^	96 ± 16 ^ab^	114 ± 17 ^bc^	72 ± 8 ^a^
PetB (cyt b_6_)	100 ^cd^	115 ± 4 ^ef^	80 ± 4 ^b^	88 ± 5 ^bc^	89 ± 4 ^bc^	60 ± 3 ^a^
PetC (Rieske)	100 ^b^	152 ± 9 ^d^	122 ± 6 ^c^	130 ± 5 ^c^	106 ± 7 ^b^	85 ± 2 ^a^
PsbA (D1)	100 ^ab^	94 ± 15 ^a^	113 ± 15 ^abc^	141 ± 17 ^cde^	140 ± 14 ^cde^	145 ± 14 ^de^
PsbD (D2)	100 ^de^	86 ± 3 ^bcd^	57 ± 5 ^a^	50 ± 5 ^a^	76 ± 5 ^b^	82 ± 5 ^bc^
PsbC	100 ^e^	78 ± 3 ^d^	36 ± 3 ^a^	41 ± 5 ^ab^	80 ± 6 ^d^	50 ± 4 ^bc^
PsbB	100 ^f^	60 ± 6 ^c^	34 ± 4 ^a^	42 ± 5 ^ab^	66 ± 7 ^cd^	58 ± 12 ^bc^
Lhcb1	100 ^e^	86 ± 4 ^cd^	99 ± 5 ^e^	95 ± 6 ^de^	132 ± 5 ^f^	78 ± 4 ^bc^
Lhcb2	100 ^a^	121 ± 4 ^cd^	97 ± 9 ^a^	112 ± 9 ^abc^	111 ± 9 ^abc^	103 ± 5 ^ab^
Lhcb3	100 ^d^	108 ± 3 ^e^	80 ± 2 ^a^	88 ± 4 ^bc^	123 ± 3 ^f^	91 ± 2 ^bc^
Lhcb4	100 ^cd^	106 ± 2 ^d^	83 ± 2 ^a^	92 ± 2 ^b^	93 ± 2 ^bc^	102 ± 4 ^d^
Lhcb5	100 ^f^	82 ± 2 ^e^	71 ± 1 ^cd^	64 ± 2 ^c^	55 ± 1 ^ab^	61 ± 3 ^bc^
Lhcb6	100 ^e^	88 ± 2 ^d^	74 ± 4 ^bc^	80 ± 1 ^c^	71 ± 6 ^b^	63 ± 5 ^a^
PsbO	100 ^ef^	87 ± 2 ^bcd^	77 ± 4 ^abc^	71 ± 4 ^a^	74 ± 7 ^ab^	79 ± 7 ^abcd^
PsbQ	100 ^f^	158 ± 3 ^h^	51 ± 1 ^b^	85 ± 2 ^de^	50 ± 2 ^b^	80 ± 2 ^cd^
PsbS	100 ^a^	115 ± 2 ^b^	98 ± 3 ^a^	117 ± 10 ^b^	155 ± 7 ^cd^	163 ± 5 ^d^

## Data Availability

All data are contained within the article.

## Supplementary Materials

The following supporting information can be downloaded at: https://www.mdpi.com/article/10.3390/plants11172185/s1, Figure S1–S6: Figure S1: Changes in the thylakoid complexes of *Haberlea rhodopensis* during recovery from drought- (RAD) and freezing-induced desiccation (RAF); Figure S2: 2D BN/SDS PAGE pattern of thylakoids after 7 d recovery; Figure S3: Representative Western blots of the main thylakoid-related proteins of *Haberlea rhodopensis* during the recovery from drought- (RAD) and freezing-induced desiccation (RAF); Figure S4: In-gel activity staining of protease bands at pH 6.0 and pH 8.5 during the first hours of RAD and RAF; Figure S5: Multiple alignment of ELI_PEA protein (acc. No SP:P11432) with the translated open reading frames (ORFs) encoded by the *ELIP* encoding contigs, identified by the BLAST search [85]; Figure S6: Expression heat map of the identified contigs, encoding ELIP proteins under drought, desiccation and rehydration based on published RNAseq data. Table S1: Primers used in qRT-PCR analysis. Data S1: Local TBLASTN output of Pea ELIP protein against the published *Haberlea rhodopensis* RNAseq contigs database [86]; References: [18,41].
