# Supplementary material for: Reactivation of the Photosynthetic Apparatus of Resurrection Plant Haberlea rhodopensis during the Early Phase of Recovery from Drought- and Freezing-Induced Desiccation
Source: Plants (Basel). 2022 Aug 23;11(17):2185. doi: 10.3390/plants11172185 (PMC9460447; doi:10.3390/plants11172185)
Supplement: Supplementary file 1 [file plants-11-02185-s001.zip › plants-1846549-supplementary Data S1.pdf]

Supplementary material

Reactivation of the photosynthetic apparatus of resurrection plant *Haberlea rhodopensis* during the early phase of recovery from drought- and freezing-induced desiccation

Gergana Mihailova [1], Nikolai K. Christov [2], Éva Sárvári [3], Ádám Solti [3], Richard Hembrom [4], Katalin Solymosi [4], Áron Keresztes [4], Maya Velitchkova [5], Antoaneta V. Popova [5], Lyudmila Simova-Stoilova [1], Elena Todorovska [2] and Katya Georgieva [1,*]

[1] Institute of Plant Physiology and Genetics, Bulgarian Academy of Sciences, Acad. G. Bonchev Str., Bl. 21, 1113 Sofia, Bulgaria
[2] AgroBioInstitute, Agricultural Academy, 8 Dragan Tsankov Blvd., 1164 Sofia, Bulgaria
[3] Department of Plant Physiology and Molecular Plant Biology, Institute of Biology, Faculty of Science, ELTE Eötvös Loránd University, Pázmány P. sétány 1/C, H-1117 Budapest, Hungary
[4] Department of Plant Anatomy, Institute of Biology, Faculty of Science, ELTE Eötvös Loránd University, Pázmány P. sétány 1/C, Budapest 1117, Hungary
[5] Institute of Biophysics and Biomedical Engineering, Bulgarian Academy of Sciences, Acad. G. Bonchev Str., Bl. 21, 1113 Sofia, Bulgaria
* Correspondence: katya@bio21.bas.bg or georgieva.katya.m@gmail.com; Tel.: +359-2-979-2620

Data S1. Local TBLASTN output of Pea ELIP protein against the published *Haberlea rhodopensis* RNAseq contigs database [41]. A cut-off E-value of 1e^{-05} was applied to limit the output to contigs encoding the most similar proteins. We named the identified contigs according to homology of the encoded proteins to the pea ELIP used as the query. The annotated names are highlighted in the pair-wise alignments.

```
TBLASTN 2.2.26 [Sep-21-2011]


Reference: Altschul, Stephen F., Thomas L. Madden, Alejandro A. Schaffer,
Jinghui Zhang, Zheng Zhang, Webb Miller, and David J. Lipman (1997),
"Gapped BLAST and PSI-BLAST: a new generation of protein database search
programs",  Nucleic Acids Res. 25:3389-3402.

Reference for compositional score matrix adjustment: Altschul, Stephen F.,
John C. Wootton, E. Michael Gertz, Richa Agarwala, Aleksandr Morgulis,
Alejandro A. Schaffer, and Yi-Kuo Yu (2005) "Protein database searches
using compositionally adjusted substitution matrices", FEBS J. 272:5101-5109.

Query= sp|P11432|ELI_PEA Early light-induced protein, chloroplastic
OS=Pisum sativum OX=3888 PE=2 SV=1
         (196 letters)

Database: TG_Hrh_contigs.fasta
           96,353 sequences; 40,586,049 total letters

Searching..................................................done



                                                                 Score    E
Sequences producing significant alignments:                      (bits) Value

>Contig_003481                                                    126   6e-36
>Contig_093673                                                    125   6e-36
>Contig_024549                                                     83   4e-20
>Contig_093552                                                     48   1e-07

>>Contig_003481 ELIP1
          Length = 459

 Score =  126 bits (316), Expect = 6e-36, Method: Compositional matrix adjust.
 Identities = 60/111 (54%), Positives = 74/111 (66%)
 Frame = -1

Query: 86  KFSDLMAFSGPAPERINGRLAMIGFVAAMGVEIAKXXXXXXXXXXXXXVAWFLGTSVLLSL 145
           K +D+MAF GP PERINGRLAMIGFVA + VE+              + WF+GT+VLLS+
Sbjct: 459 KITDIMAFDGPGPERINGRLAMIGFVADIAVELTNGQDIFSQIQNGGIPWFIGTTVLLSI 280

Query: 146 ASLIPFFQGXXXXXXXXXXXXXDAEFWNGRIAMLGLVALAFTEFVKGTSLV 196
           ASL+P F+G             DAE WNGR AMLGL+ALA+TE+VKG +LV
Sbjct: 279 ASLVPLFKGVSADSKSGELMTSDAELWNGRFAMLGLIALAYTEYVKGGALV 127


>>Contig_093673 ELIP2
          Length = 437
```

```
Score =  125 bits (315),Expect = 6e-36, Method: Compositional matrix adjust.
Identities = 59/110 (53%), Positives = 73/110 (66%)
Frame = +3

Query: 81  PKMSTKFSDLMAFSGPAPERINGRLAMIGFVAAMGVEIAKXXXXXXXXXXXXVAWFLGTS 140
           P+ STK +D+MAF GP PERINGRLAMIGFVAA+ VE+ +            + WFLGT
Sbjct: 6   PEESTKITDIMAFDGPGPERINGRLAMIGFVAAIAVELTRGHDIFTQIQNGGIPWFLGTI 185

Query: 141 VLLSLASLIPFFQGXXXXXXXXXXXXXDAEFWNGRIAMLGLVALAFTEFV 190
           VLLS+ SL+P F+G             DAE WNGR AMLGL+ALA+TE++
Sbjct: 186 VLLSITSLVPLFKGVSADSKSGGVMTSDAELWNGRFAMLGLIALAYTEYI 335


>>Contig_024549 ELIP3
          Length = 354

Score = 83.2 bits (204), Expect = 4e-20, Method: Compositional matrix adjust.
Identities = 40/84 (47%), Positives = 53/84 (63%)
Frame = -3

Query: 113 AMGVEIAKXXXXXXXXXXXXVAWFLGTSVLLSLASLIPFFQGXXXXXXXXXXXXXDAEFW 172
           A+ VE+ K            ++WF+GT+VLLS+ASL+P F+G             DAE W
Sbjct: 352 AIAVELTKGQDIFSQIQNGGISWFIGTTVLLSVASLVPLFKGVSADSKSGGLMTSDAELW 173

Query: 173 NGRIAMLGLVALAFTEFVKGTSLV 196
           NGR AMLGL+ALA+TE+VKG +LV
Sbjct: 172 NGRFAMLGLIALAYTEYVKGGTLV 101


>>Contig_093552 ELIP4
          Length = 256

Score = 47.8 bits (112), Expect = 1e-07, Method: Compositional matrix adjust.
Identities = 20/29 (68%), Positives = 25/29 (86%)
Frame = +1

Query: 168 DAEFWNGRIAMLGLVALAFTEFVKGTSLV 196
           DAE WNGR+AM+GLV LA+TE++KG  LV
Sbjct: 154 DAELWNGRLAMVGLVELAYTEYLKGGPLV 240




Database: TG_Hrh_contigs.fasta
Posted date:  Jul 31, 2020 12:56 PM
  Number of letters in database: 40,586,049
  Number of sequences in database:  96,353

Lambda     K      H
   0.322    0.134    0.389

Gapped
Lambda     K      H
   0.267   0.0410    0.140
```

```
Matrix: BLOSUM62
Gap Penalties: Existence: 11, Extension: 1
Number of Sequences: 96353
Number of Hits to DB: 5,472,580
Number of extensions: 40379
Number of successful extensions: 157
Number of sequences better than 10.0: 11
Number of HSP's gapped: 154
Number of HSP's successfully gapped: 15
Length of query: 196
Length of database: 13,528,683
Length adjustment: 90
Effective length of query: 106
Effective length of database: 4,856,913
Effective search space: 514832778
Effective search space used: 514832778
Neighboring words threshold: 13
Window for multiple hits: 40
X1: 16 ( 7.4 bits)
X2: 38 (14.6 bits)
X3: 64 (24.7 bits)
S1: 41 (22.0 bits)
S2: 1 ( 5.0 bits)
```
