# Supplementary material for: Reactivation of the Photosynthetic Apparatus of Resurrection Plant Haberlea rhodopensis during the Early Phase of Recovery from Drought- and Freezing-Induced Desiccation
Source: Plants (Basel). 2022 Aug 23;11(17):2185. doi: 10.3390/plants11172185 (PMC9460447; doi:10.3390/plants11172185)
Supplement: Supplementary file 1 [file plants-11-02185-s001.zip › plants-1846549-supplementary Figures.pdf]

Supplementary material

Reactivation of the photosynthetic apparatus of resurrection plant *Haberlea rhodopensis* during the early phase of recovery from drought- and freezing-induced desiccation

Gergana Mihailova [1], Nikolai K. Christov [2], Éva Sárvári [3], Ádám Solti [3], Richard Hembrom [4], Katalin Solymosi [4], Áron Keresztes [4], Maya Velitchkova [5], Antoaneta V. Popova [5], Lyudmila Simova-Stoilova [1], Elena Todorovska [2] and Katya Georgieva [1,*]

[1] Institute of Plant Physiology and Genetics, Bulgarian Academy of Sciences, Acad. G. Bonchev Str., Bl. 21, 1113 Sofia, Bulgaria
[2] AgroBioInstitute, Agricultural Academy, 8 Dragan Tsankov, 1164 Sofia, Bulgaria
[3] Department of Plant Physiology and Molecular Plant Biology, Institute of Biology, Faculty of Science, ELTE Eötvös Loránd University, Pázmány P. sétány 1/C, H-1117 Budapest, Hungary
[4] Department of Plant Anatomy, Institute of Biology, Faculty of Science, ELTE Eötvös Loránd University, Pázmány P. sétány 1/C, Budapest 1117, Hungary
[5] Institute of Biophysics and Biomedical Engineering, Bulgarian Academy of Sciences, Acad. G. Bonchev Str., Bl. 21, 1113 Sofia, Bulgaria
* Correspondence: katya@bio21.bas.bg or georgieva.katya.m@gmail.com; Tel.: +359-2-979-2620

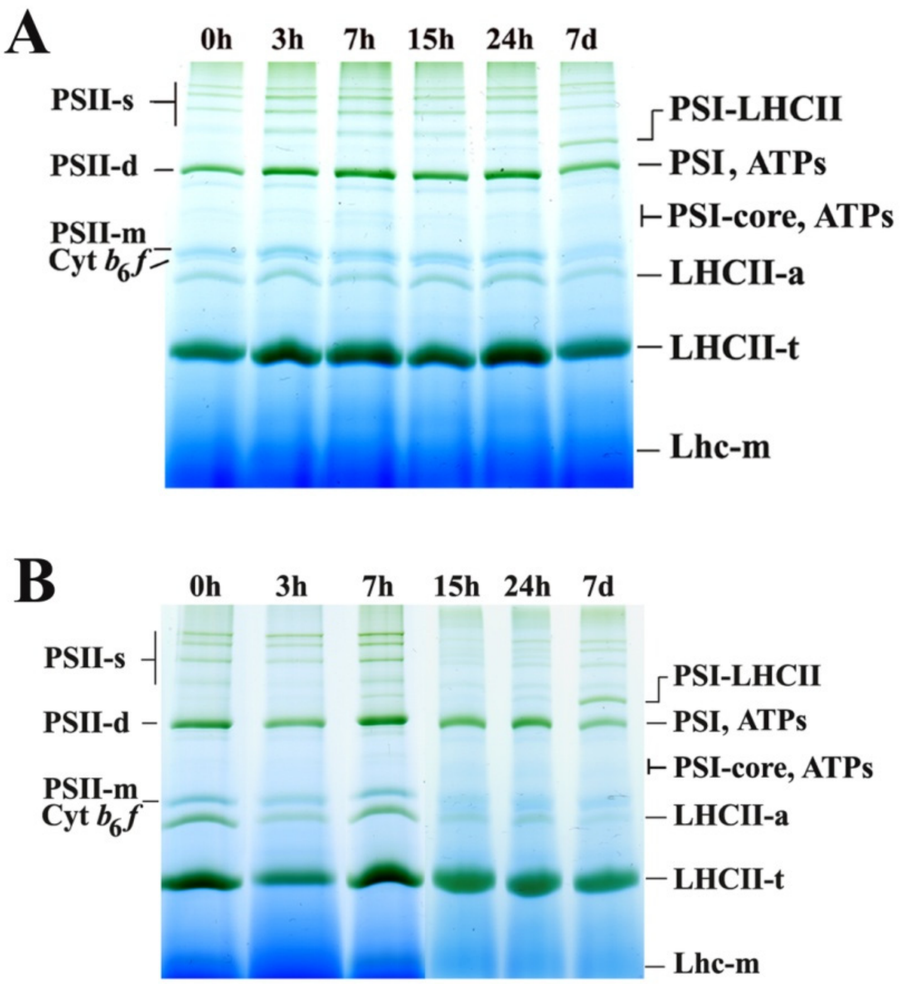

Figure S1. Changes in the thylakoid complexes of *Haberlea rhodopensis* during recovery from drought- (RAD; A) and freezing-induced desiccation (RAF; B). Thylakoids (500 µg Chl mL^{-1}) were solubilised using 1% (w/V) *β*-DM plus 1% (w/V) digitonin, and complexes were separated in 4.3–12% Blue Native (BN) gel gradients. PS—photosystem; Cyt—cytochrome; ATPs—ATP synthase; LHC/Lhc—light-harvesting complex; LHCII-a—LHCII assembly: CP29 + CP24 + LHCII-t; CP—chlorophyll-protein; s—supercomplex; t—trimer; d—dimer; m—monomer.

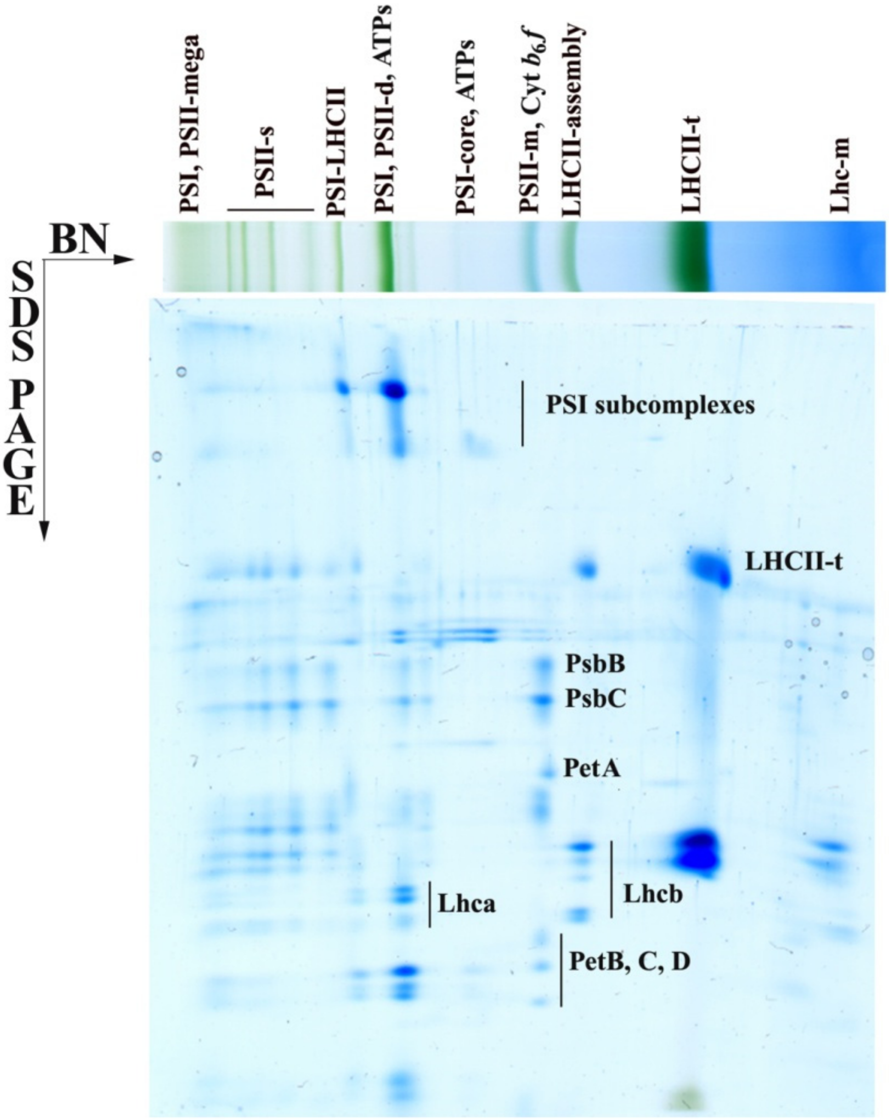

Figure S2. 2D BN/SDS PAGE pattern of thylakoids after 7 d recovery. Thylakoids (500 µg Chl mL^{-1}) were solubilised using 1% (w/V) β-DM plus 1% (w/V) digitonin, and complexes were separated in 4.3–12% BN gel gradients followed by SDS PAGE. Complexes are marked as in Figure S1.

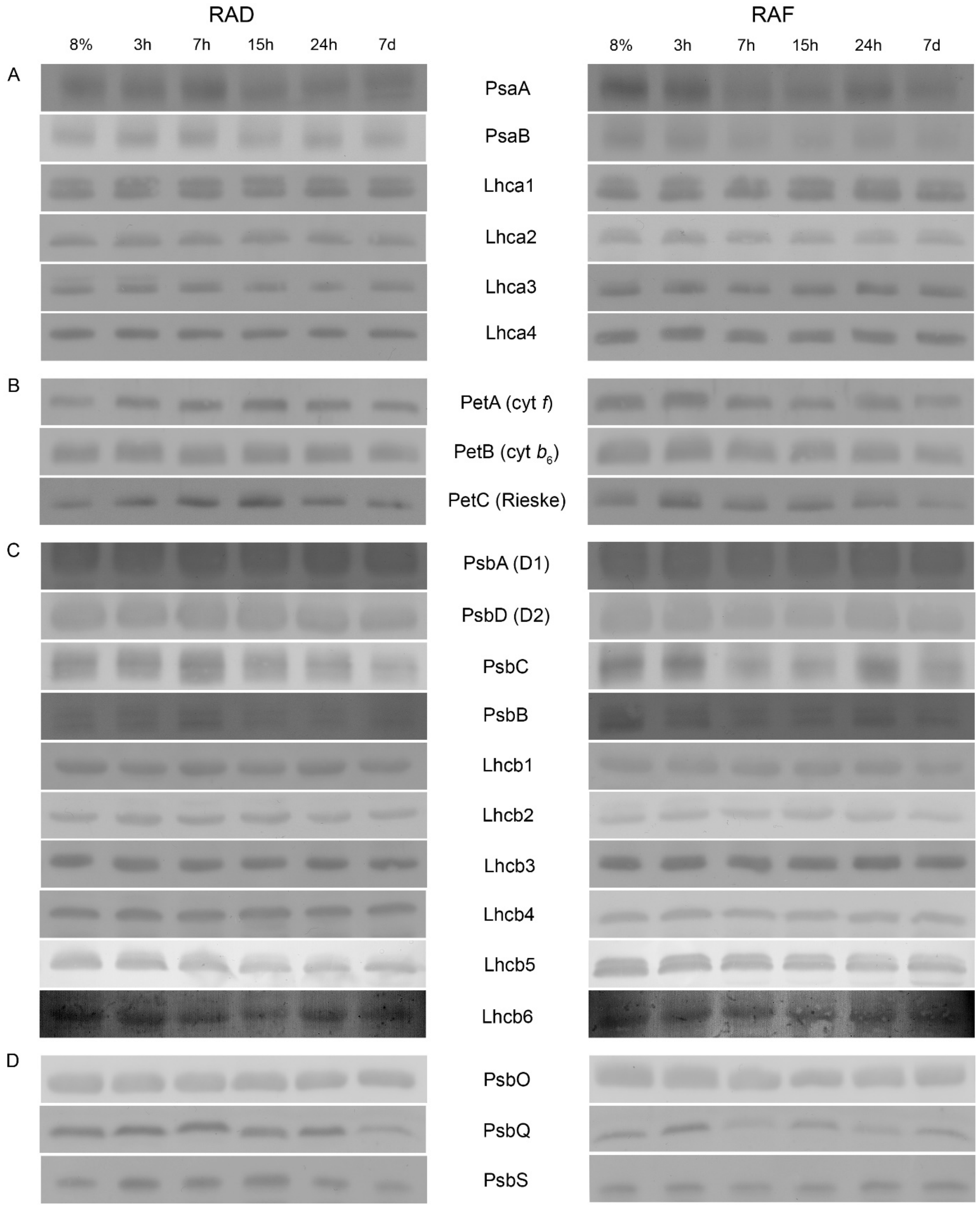

Figure S3. Representative Western blots of the main thylakoid-related proteins of *Haberlea rhodopensis* during the recovery from drought- (RAD) and freezing-induced desiccation (RAF). 8%—dried plant; 3, 7, 15 and 24 h—hours after rehydration; 7 d—7 days after rehydration.

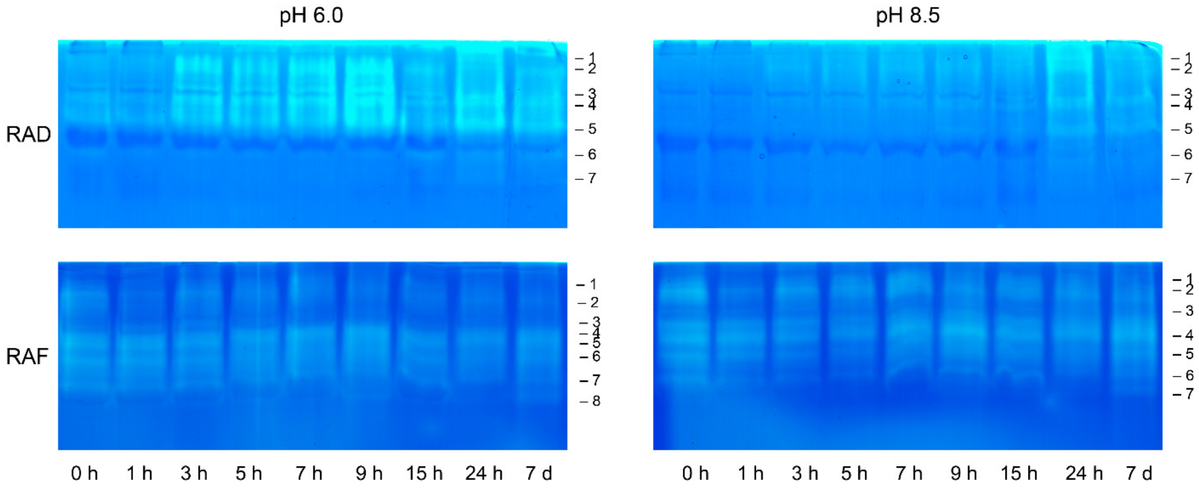

Figure S4. In-gel activity staining of protease bands at pH 6.0 (left) and pH 8.5 (right) during the first hours of RAD (top images) and RAF (bottom images). 8%—dried plant; 1, 3, 5, 7, 9, 15 and 24 h—hours after rehydration; 7 d—7 days after rehydration. Activity bands are numbered in order of increasing electrophoretic mobility.

```
                  *        20         *        40         *        60
ELI_PEA : MAVSSCQSIMSNSMTNISSRSRVNQFTNIPSVYIPTLRRNVSLKVRSMAEGEPKEQSKVA :  60
ELIP1   : ------------------------------------------------------------ :   -
ELIP2   : ------------------------------------------------------------ :   -
ELIP3   : ------------------------------------------------------------ :   -
ELIP4   : ------------------------------------------------------------ :   -

                  *        80         *       100         *       120
ELI_PEA : VDPTTPTASTPTPQPAYTRPPKMSTKFSDLMAFSGPAPERINGRLAMIGFVAAMGVEIAK : 120
ELIP1   : -------------------------KITDIMAFDGPGPERINGRLAMIGFVADIAVELTN :  35
ELIP2   : -------------------QPEESTKITDIMAFDGPGPERINGRLAMIGFVAAIAVELTR :  41
ELIP3   : ----------------------------------------------------AIAVELTK :   8
ELIP4   : ------------------------------------------------------------ :   -

                  *       140         *       160         *       180
ELI_PEA : GQGLSEQLSGGGVAWFLGTSVLLSLASLIPFFQGVSVESKSKSIMSSDAEFWNGRIAMLG : 180
ELIP1   : GQDIFSQIQNGGIPWFIGTTVLLSIASLVPLFKGVSADSKSGELMTSDAELWNGRFAMLG :  95
ELIP2   : GHDIFTQIQNGGIPWFLGTIVLLSITSLVPLFKGVSADSKSGGVMTSDAELWNGRFAMLG : 101
ELIP3   : GQDIFSQIQNGGISWFIGTTVLLSVASLVPLFKGVSADSKSGGLMTSDAELWNGRFAMLG :  68
ELIP4   : --------------------AVLSVASLIPLFKGISADLKSGGFFKSDAELWNGRLAXXX :  40

                  *       200         *
ELI_PEA : LVALAFTE------FVKGTSLV------------- : 196
ELIP1   : LIALAYTE------YVKGGALV------------- : 111
ELIP2   : LIALAYTE------YITQGWYSCVKGFVRLFLVP- : 129
ELIP3   : LIALAYTE------YVKGGTLV------------- :  84
ELIP4   : XIRNPEDFLNLMQSFGMGG-LP-WSGLWNWLILSI :  73
```

Figure S5. Multiple alignment of ELI_PEA protein (acc. No SP:P11432) with the translated open reading frames (ORFs) encoded by the ELIP encoding contigs, identified by the BLAST search. The ORFs were identified and translated by the NCBI orffinder tool (https://www.ncbi.nlm.nih.gov/orffinder/) and aligned using Clustal Omega [85].

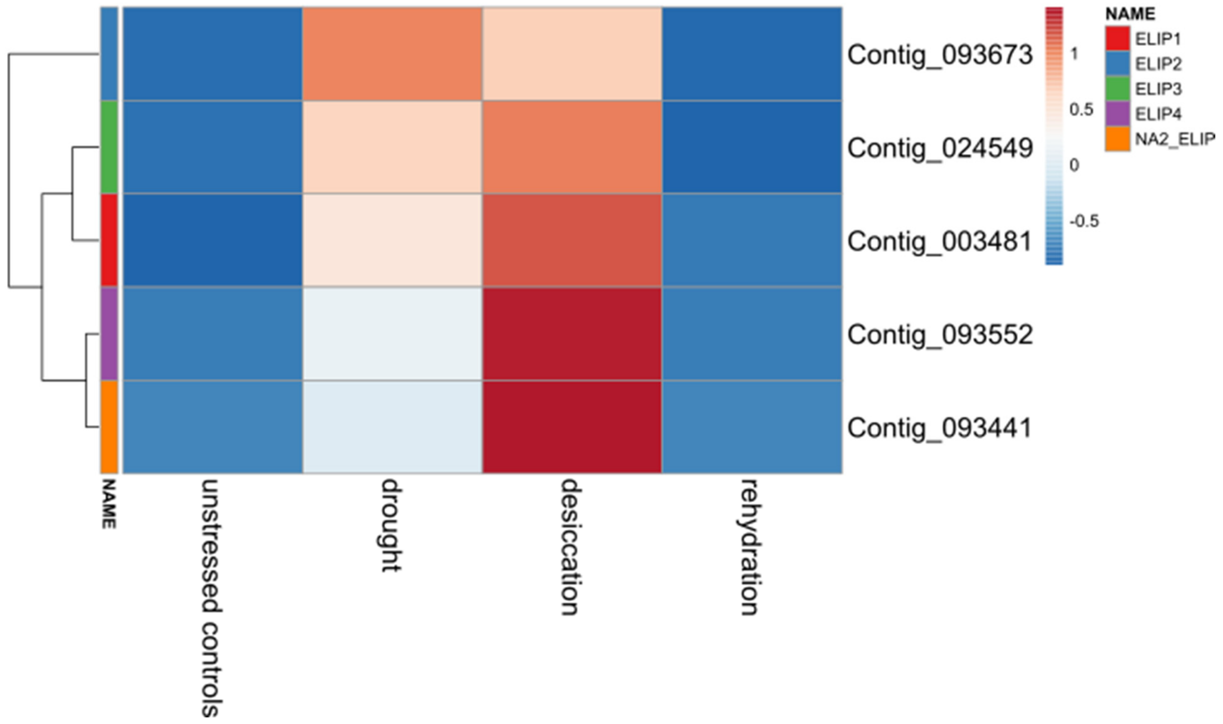

Figure S6. Expression heat map of the identified contigs, encoding ELIP proteins under drought, desiccation and rehydration based on published RNAseq data [41].
